# Integrated prelithiation and SEI engineering for high-performance silicon anodes in lithium-ion batteries

**DOI:** 10.1093/nsr/nwaf084

**Published:** 2025-03-03

**Authors:** Lijiao Quan, Qili Su, Haozhe Lei, Wenguang Zhang, Yingkang Deng, Jiarong He, Yong Lu, Zhe Li, Haijing Liu, Lidan Xing, Weishan Li

**Affiliations:** School of Chemistry, South China Normal University, Guangzhou 510006, China; National and Local Joint Engineering Research Center of MPTES in High Energy and Safety LIBs, Engineering Research Center of MTEES (Ministry of Education), Research Center of BMET (Guangdong Province), and Key Laboratory of ETESPG(GHEI), South China Normal University, Guangzhou 510006, China; Battery Research and Development, Battery Propulsion & Sustainability, General Motors, Shanghai 201206, China; School of Chemistry, South China Normal University, Guangzhou 510006, China; School of Chemistry, South China Normal University, Guangzhou 510006, China; National and Local Joint Engineering Research Center of MPTES in High Energy and Safety LIBs, Engineering Research Center of MTEES (Ministry of Education), Research Center of BMET (Guangdong Province), and Key Laboratory of ETESPG(GHEI), South China Normal University, Guangzhou 510006, China; School of Chemistry, South China Normal University, Guangzhou 510006, China; National and Local Joint Engineering Research Center of MPTES in High Energy and Safety LIBs, Engineering Research Center of MTEES (Ministry of Education), Research Center of BMET (Guangdong Province), and Key Laboratory of ETESPG(GHEI), South China Normal University, Guangzhou 510006, China; School of Chemistry, South China Normal University, Guangzhou 510006, China; National and Local Joint Engineering Research Center of MPTES in High Energy and Safety LIBs, Engineering Research Center of MTEES (Ministry of Education), Research Center of BMET (Guangdong Province), and Key Laboratory of ETESPG(GHEI), South China Normal University, Guangzhou 510006, China; Battery Research and Development, Battery Propulsion & Sustainability, General Motors, Shanghai 201206, China; Battery Research and Development, Battery Propulsion & Sustainability, General Motors, Shanghai 201206, China; Battery Research and Development, Battery Propulsion & Sustainability, General Motors, Shanghai 201206, China; School of Chemistry, South China Normal University, Guangzhou 510006, China; National and Local Joint Engineering Research Center of MPTES in High Energy and Safety LIBs, Engineering Research Center of MTEES (Ministry of Education), Research Center of BMET (Guangdong Province), and Key Laboratory of ETESPG(GHEI), South China Normal University, Guangzhou 510006, China; School of Chemistry, South China Normal University, Guangzhou 510006, China; National and Local Joint Engineering Research Center of MPTES in High Energy and Safety LIBs, Engineering Research Center of MTEES (Ministry of Education), Research Center of BMET (Guangdong Province), and Key Laboratory of ETESPG(GHEI), South China Normal University, Guangzhou 510006, China

**Keywords:** amorphous silicon, chemical prelithiation, Li-Naph, solid electrolyte interphase, battery performance

## Abstract

Improving the initial Coulombic efficiency (ICE) of silicon anodes in lithium-ion batteries is a key challenge for enhancing their performance. Traditional prelithiation methods, such as using lithium naphthalenide (Li-Naph), are limited by the low lithiation potential of crystalline silicon, making them less effective for commercial applications. This study demonstrates that amorphous silicon anodes, with a higher lithiation potential, can be effectively prelithiated using Li-Naph. This prelithiation process also forms a robust solid electrolyte interphase, which significantly enhances the anode's cycling stability and overall battery performance. The prelithiated silicon anodes achieved a remarkable ICE improvement from 74.8% to 97.2% in full-cell tests. Furthermore, 27 mAh pouch cells exhibited excellent long-cycle stability and low-temperature performance, retaining 90.1% of their capacity after 800 cycles at 1 C. These findings highlight the potential for scalable prelithiation methods and open new avenues for advancing silicon anode technology in next-generation batteries.

## Teaser

A scalable prelithiation strategy not only significantly improves the initial efficiency of amorphous silicon anodes, but also promotes the formation of a robust SEI, enabling enhanced cycling stability and high-performance lithium-ion pouch cells.

## INTRODUCTION

Solid-state and semi-solid-state batteries have garnered significant attention in both academia and industry in recent years, primarily due to their exceptional safety, remarkable power density and environmental friendliness [1–3]. The escalating demand for batteries with high energy density necessitates anode materials with superior specific capacity beyond graphite, prompting a focus on alternatives such as lithium metal and silicon anodes. The lithium metal anode, exhibiting a 10-fold increase in specific capacity compared to graphite (3860 mAh g^−1^) [4,5], presents significant potential for high-energy-density batteries. However, practical applications are challenged by the inherent tendency of lithium dendrite formation and its high reactivity with electrolytes, leading to safety hazards and performance limitations [6,7]. Silicon (Si) materials have gained widespread attention due to their impressive specific capacity (3580 mAh g^−1^ for Li_15_Si_4_) and slightly higher lithiation potential (0.30 V higher than Li), which effectively mitigate the formation of lithium dendrites and showcase immense potential for achieving high-energy-density batteries [8,9]. Moreover, the abundant presence of Si in nature, coupled with its environmentally friendly and highly safe characteristics, renders it an ideal candidate for anode materials.

However, Si materials face a significant challenge in their low initial Coulombic efficiency (ICE), attributed to the substantial consumption of active lithium caused by the formation of the solid electrolyte interphase (SEI) and lithium trapping within the bulk materials [10–12]. This results in a subsequent net capacity loss, severely limiting the available energy density of batteries. To address this issue, various strategies have been developed, including prelithiating Si anodes to compensate for the initial lithium loss and enable full utilization of lithium sources within the cathode while maximizing overall battery energy density. Currently, techniques for prelithiation of anodes can be broadly categorized into four groups: direct contact with lithium foil or stable lithium metal powder (SLMP), prelithiation assisted by active materials, electrochemical prelithiation and chemical prelithiation [13,14]. Direct contact between the anode and lithium foil prompts an automatic transfer of electrons from the lithium foil to the anode, driven by the potential difference across the electrodes. This electron transfer is accompanied by the migration of lithium ions, ensuring the electrical neutrality of the materials. However, this method lacks control over both prelithiation degree and uniformity and necessitates a controlled environment due to the sensitivity of lithium foil to air [10]. To mitigate the sensitivity of lithium metal to air, SLMP coated with air-stabilized Li_2_CO_3_ has been developed, enabling effective prelithiation of anode materials in ambient environments [15]. Nevertheless, achieving homogeneity in prelithiation remains challenging due to the large particle size of SLMP (5–50 μm). Additionally, an additional pressure activation process is typically required to rupture the Li_2_CO_3_ shell and ensure sufficient contact between the anode and lithium metal [16]. Active material-assisted prelithiation involves the utilization of compounds such as LiOH [17], LiH [18], LiBH_4_ [19] and Li_x_Si@Li_2_O [20]. However, compatibility issues may emerge between these active substances and various battery components, including binders, solvents and electrolytes [14]. Identifying a suitable prelithiation reagent continues to pose a challenge. Electrochemical prelithiation offers precise control over the degree of prelithiation through voltage manipulation in a temporary half-cell. However, the cumbersome disassembly/reassembly procedures associated with batteries hinder its industrial or commercial viability, thereby limiting its application primarily to laboratory research.

Chemical prelithiation, driven by the redox potential difference between the lithiation reagent and the target electrode, offers simplicity, homogeneity and efficiency [13]. Its straightforward operational procedures and precise control over lithiation degree make it more conducive to widespread industrial adoption compared to alternative methods. Lithiation reagent solutions utilized in chemical prelithiation typically comprise a highly reductive chemical (Li-arene) dissolved in a chemically stable solvent (such as dimethoxy ethane (DME) or tetrahydrofuran (THF)). Two prominent lithiation reagents, Li-naphthalene/DME (Li-Naph) [21] and Li-biphenyl/THF (Li-BP) [22], effectively and controllably lithiate S cathodes [23], P anodes [24] and hard carbon anodes [25] to desired levels by optimizing reaction time, benefiting from their mild reactivity and robust lithiation capabilities. Unfortunately, these reagents have thus far proven inadequate for doping active Li into Si-based anodes, as the initial lithiation potential of Si anodes (<0.20 V vs. Li^+^/Li) falls below the redox potential of both Li-Naph [23,25] and Li-BP (∼0.33 V vs. Li^+^/Li) [26] reagents. Consequently, current efforts are focused on developing chemical reagents with sufficient reducing strength to facilitate effective chemical prelithiation of Si-based anodes [27–29].

Upon closely scrutinizing the charge-discharge profiles of silicon anodes, it is apparent that except for the initial charging cycle, the lithiation voltage in subsequent charging processes reaches ∼0.45 V, surpassing the voltages associated with the mentioned lithiation reagents [30–32]. This implies that by comprehending the factors contributing to the disparity in lithiation voltages between the initial and subsequent cycles of Si anodes and elevating the lithiation voltage during the initial cycle to align with that of subsequent cycles, we can overcome the obstacle hindering traditional chemical prelithiation's application to Si anodes. Regarding the change in lithiation potential of Si anodes after the first charge-discharge cycle, it is currently believed that this primarily results from the transformation of crystalline Si into amorphous Si following the initial lithiation reaction [11,33]. Concurrently, some researchers suggest that residual lithium in the Si anode after the initial cycle may also contribute to the increased lithiation voltage in subsequent cycles [34,35]. Thus, the precise factor behind the change in lithiation potential for silicon anodes remains unclear. Despite these insights, there have been no reports to date on studies that adjust the initial lithiation potential of Si anodes to match the electrochemical window of traditional prelithiation reagents.

Herein, this study systematically investigates the critical influence of silicon material structure on its initial lithiation potential and further validates this concept using crystalline silicon materials and amorphous columnar silicon film (col-Si) prepared by the physical vapor deposition (PVD) method. The experimental results indicate that Li-Naph and Li-BP reagents are ineffective in prelithiating crystalline Si, while they can effectively prelithiate amorphous col-Si. Full coin cells were assembled utilizing prelithiated col-Si anodes and composite cathodes comprising LiMn_2_O_4_ (LMO) and LiMn_0.7_Fe_0.3_PO_4_ (LMFP) in a semi-solid-state electrolyte based on fluoroethylene carbonate (FEC) and gamma-butyrolactone (GBL). The ICE of these full-cells exhibited a significant increase from 74.8% to 97.2%, thereby enhancing the utilization of active lithium within the cathode materials. More importantly, this prelithiation strategy reduces the initial electrode potential of Si, promoting simultaneous reduction and decomposition of electrolyte components on the Si anode surface. This results in the formation of a robust, uniformly distributed SEI film rich in LiF and boron compounds, significantly enhancing the cycling stability of Si. Additionally, prelithiation greatly improves lithium-ion utilization, allowing for most lithium ions to be re-intercalated into the cathode material. This effectively prevents some cathode materials from remaining in a high-voltage delithiated state, thereby inhibiting dissolution of transition metal ions and reducing capacity degradation. Subsequently, this technology was scaled up to 27 mAh pouch cells, which exhibited notable cycling performance and excellent cold-cranking capabilities. After undergoing 800 cycles at 25°C with a current rate of 1 C, the pouch cells retained 90.1% of their capacity, thus demonstrating outstanding durability and surpassing the results documented in prior investigations (Table S1). Furthermore, the pouch cells demonstrated effective operation at −18°C with a high current density of 10 C.

This study demonstrates a breakthrough in enhancing silicon anode performance by effectively integrating prelithiation with SEI engineering. The approach significantly boosts initial Coulombic efficiency and cycling stability, with successful application in scaled-up pouch cells. This scalable strategy offers promising potential for industrial applications, providing a pathway for the commercialization of high-performance silicon-based anodes in next-generation lithium-ion batteries (LIBs).

## RESULTS

### Factors influencing the initial lithiation potential of silicon anodes

**Figure 1.**
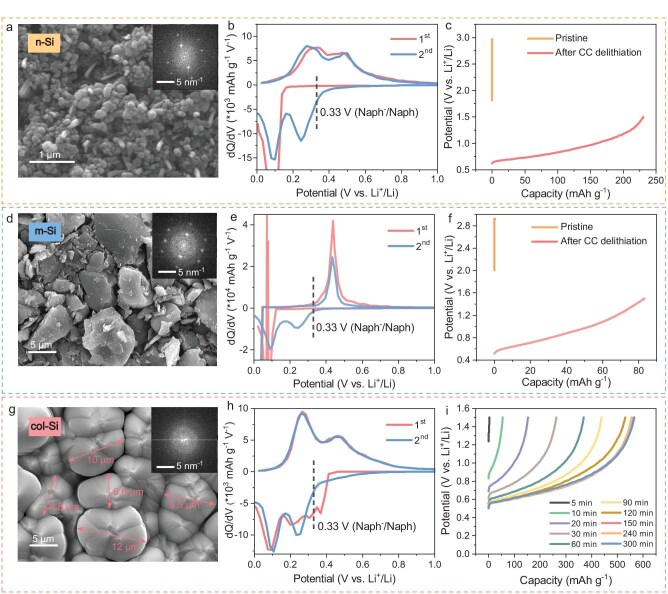
Structure and electrochemical behavior of different silicon materials. SEM images (insets: fast Fourier transform images from TEM) of (a) n-Si, (d) m-Si and (g) col-Si. Differential capacity (dQ/dV) profiles during the initial two cycles of half-cells of (b) n-Si/Li, (e) m-Si/Li and (h) col-Si/Li. Delithiation process of half-cells with either pristine Si electrodes or Si electrodes after one cycle ((c) n-Si, (f) m-Si), all prelithiated in Li-Naph reagent for 150 minutes. (i) Delithiation process of half-cells with col-Si anodes after prelithiation in Li-Naph reagent for different times.

Figure 1a is the morphology of the commercial nano-sized Si (denoted by n-Si hereafter), which exhibits a crystalline structure (Fig. S1a–c). Fig. S2 and Fig. 1b present the charge-discharge profiles and corresponding dQ/dV curves for the first two cycles of n-Si/Li half-cell in a liquid electrolyte of 0.8 M LiTFSI + 0.8 M LiBF_4_-FEC/GBL (3 : 7 by weight). It is noteworthy that all Si/Li half-cells were evaluated in the same electrolyte. The initial lithiation potential of the first cycle is ∼0.18 V, notably lower than the redox potential of Li-Naph and Li-BP. In the second cycle, this potential increases to ∼0.60 V, surpassing that of the lithiation reagents. Results from X-ray diffraction (XRD), Raman spectroscopy and transmission electron microscopy (TEM, Fig. S1) confirm that n-Si undergoes a crystalline-to-amorphous transition after the first cycle, consistent with previous reports [36,37]. Figure 1c shows the delithiation performance of half-cells assembled with lithium foil and either pristine n-Si anodes or n-Si anodes after one cycle, both soaked in Li-Naph reagent for 150 minutes. As expected, the pristine n-Si/Li half-cell shows no capacity, indicating unsuccessful prelithiation. In contrast, the cycled n-Si demonstrates successful prelithiation with Li-Naph, displaying an open circuit potential (OCP) of ∼0.42 V and a delithiation capacity of ∼230.8 mAh g^−1^. To explore whether the change in lithiation potential stems from the crystalline-to-amorphous transition, the influence of residual lithium, or a combination of both, validation experiments were conducted. As depicted in Fig. S1d, residual Li-Si alloy is indeed present in the Si anode after one cycle of constant current (CC) delithiation. To ensure complete removal of residual lithium, a 1.50 V constant voltage (CV) was applied to the n-Si/Li half-cell for 12 hours post one CC cycle, and no Li-Si alloy was detected after the CCCV process (Fig. S1e). Fig. S3 presents the dQ/dV curves of Si/Li half-cells after one cycle, both with and without CV application, indicating that residual lithium has minimal impact on the lithiation potential. This reveals that the structural transition from crystalline to amorphous Si is responsible for the significant difference in the initial lithiation potentials between the first and second cycles. Concurrently, experiments conducted with commercial micron-sized silicon anodes (m-Si) show similar results (Fig. 1d–f and Fig. S4), indicating that particle size has negligible impact on the initial lithiation potential. These findings further demonstrate that the key factor influencing the difference in the initial lithiation potential of silicon anodes is their initial structural state (crystalline vs. amorphous), rather than the particle size.

To further verify the impact of Si structure on the initial lithiation potential, we fabricated an amorphous columnar Si film electrode (col-Si, Fig. S5) using the physical vapor deposition (PVD) method. The top-view scanning electron microscopy (SEM) image (Fig. 1g) shows that col-Si particles on a micron scale are closely packed. Fig. S6 demonstrates robust adhesion between the silicon pillars and the copper foil, with a film thickness (or pillar height) of ∼7 μm. Cross-sectional SEM and TEM mappings (Figs S6 and S7) reveal a uniform distribution of Si and oxygen elements throughout the col-Si particles. Additionally, XRD, Raman and TEM results (Fig. S8) indicate that col-Si exhibits an amorphous structure. As expected, the initial lithiation potential of col-Si rises to ∼0.41 V, higher than that of n-Si and m-Si (Fig. 1h and Fig. S9). More importantly, this potential exceeds that of the Li-Naph and Li-BP, suggesting that traditional reagents can effectively prelithiate amorphous silicon. Indeed, Fig. 1i shows the capacity released by the col-Si anodes after different soaking times in the Li-Naph solution. Unlike n-Si and m-Si (Fig. 1c and f), amorphous col-Si releases a significant capacity, which increases with soaking time until it stabilizes after 150 minutes. It is worth noting that Li-Naph was chosen for this study because literature reports indicate that its lithiation causes significantly smaller changes in electrode interfacial impedance compared to Li-BP [38], despite the higher lithiation efficiency of Li-BP (Fig. S10). To mitigate surface component influence, a detailed X-ray photoelectron spectroscopy (XPS) analysis was conducted on n-Si and col-Si. Fig. S11 reveals minimal surface disparity between the two silicon materials.

**Scheme 1.**
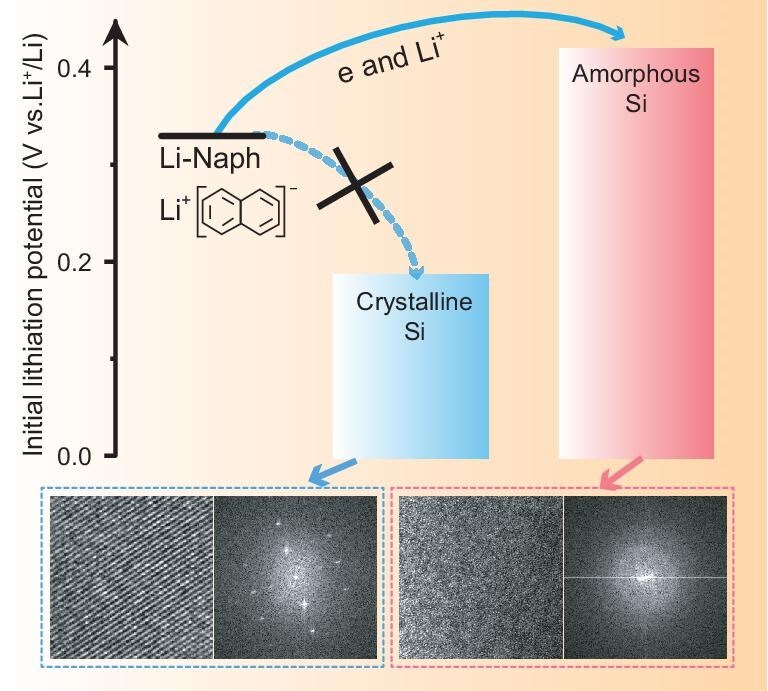
Schematic diagram of the effect of silicon structure on initial lithiation potential and prelithiation behavior.

In summary, the initial lithiation potential of Si material, critical for prelithiation feasibility with Li-Naph reagent, is predominantly determined by its structural state. As depicted in Scheme 1, lithium ions and electrons can transfer from the Li-Naph reagent to amorphous silicon with a higher lithiation potential, whereas they cannot be effectively transferred to crystalline silicon due to its lower lithiation potential.

### Prelithiation regulation of col-Si for semi-solid-state LIBs

Despite the lower electrical conductivity of col-Si anodes (which lack conductive additives) compared to n-Si and m-Si electrodes (see Table S2), col-Si exhibits enhanced cyclic stability (Fig. S12) due to its unique structural advantages. Firstly, due to the homogeneous lithiation process and the absence of severe phase transitions, amorphous Si experiences a uniform and lower stress distribution, contributing to improved structural stability [39]. Secondly, the columnar structure with intercolumnar voids effectively accommodates volume change during lithiation/delithiation, facilitating stress dispersion and mitigating the risk of material fracture [40,41]. Furthermore, the strong adhesion between silicon pillars and the roughened copper foil (Fig. S13) significantly enhances the mechanical integrity of the electrode. This minimizes material detachment and pulverization, which are commonly induced by volumetric changes during cycling. This highlights the potential of amorphous col-Si anodes for high-performance LIBs. With the challenge of low ICE in silicon-based anodes now effectively addressed by the use of traditional, straightforward and cost-efficient Li-Naph lithiation, the exploration of amorphous silicon anodes for large-scale application in LIBs holds substantial promise. Therefore, CR2025-type semi-solid-state full-cells with composite cathodes (LMO&LMFP) and prelithiated col-Si anodes were assembled and evaluated for charge-discharge performance within a voltage range of 2.50–4.20 V to assess the efficiency of prelithiation using Li-Naph reagent. The full-cells utilized a specially designed semi-solid-state electrolyte containing GBL solvent, building on our previous work, which focused on high safety standards [42–44]. The objective was to combine the high flash point and boiling point characteristics of GBL with the advantages of gel polymer electrolytes, thereby enhancing the overall safety and tolerance of the electrolyte under harsh conditions. Additionally, FEC was incorporated to enhance the durability of the Si electrode/electrolyte interphase [45]. This configuration, which combines the stability and safety of a semi-solid-state design with the simplicity and cost-effectiveness of traditional lithiation techniques, shows considerable potential for advanced LIBs applications.

**Figure 2.**
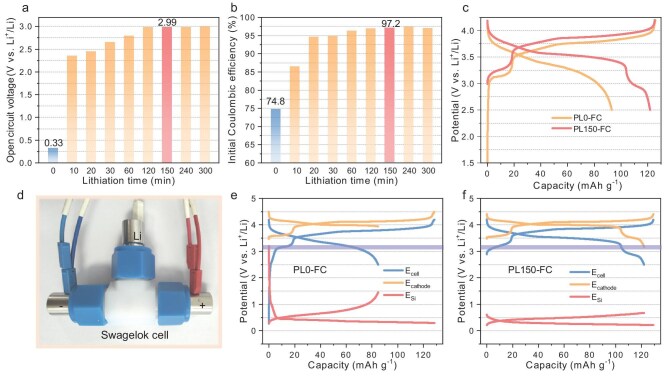
Prelithiation regulation of col-Si anodes. (a) Open circuit voltages and (b) initial Coulombic efficiency of full-cells with LMO&LMFP cathodes and col-Si anodes after prelithiation for different times. (c) The first charge-discharge profiles of full-cells with LMO&LMFP cathodes and pristine col-Si anode (PL0-FC) or col-Si after 150 minutes prelithiation (PL150-FC). (d) Photographs of a three-electrode cell and the corresponding separated voltage profiles of cathodes and anodes of (e) PL0-FC and (f) PL150-FC.

The results presented in Fig. 2a–c demonstrate that a full-cell incorporating pristine col-Si exhibits an open circuit voltage (OCV) of 0.33 V and a low ICE of 74.8%, with a charge capacity of 125.7 mAh g^−1^ and a discharge capacity of only 94 mAh g^−1^. As the prelithiation time increases from 0 to 150 minutes, both the OCV and ICE show corresponding improvements. Additionally, the characteristic peaks indicative of amorphous Si-Si bonds gradually diminish (Fig. S14), suggesting an enhanced degree of prelithiation in col-Si. After prelithiating for 150 minutes, the full-cell achieves an OCV of 2.99 V and an ICE of 97.2%, with a discharge capacity closely aligned with the theoretical specific capacity of the cathode (122 mAh g^−1^). Extending the prelithiation time to either 240 or 300 minutes leads to negligible increases in both OCV and ICE (Fig. 2a and b). Although there are no significant differences in ICE and OCV between the full-cells prelithiated for 120 minutes and those prelithiated for 150 minutes, the latter releases a higher capacity (Fig. 1i). Therefore, we selected a prelithiation time duration of 150 minutes as it strikes a balance between efficiency and performance. The col-Si anodes subjected to preliminary lithiation for 150 minutes will be referred to as PL150-Si in subsequent discussions.

Fig. S15 shows the morphology of PL150-Si, revealing that the apertures between particles have decreased due to the formation of larger-volume Li-Si alloy. Notably, in Fig. 2c, the LMO&LMFP/PL150-Si full-cell (hereafter PL150-FC) exhibits a distinct discharge plateau at ∼3.20 V, which is absent in the LMO&LMFP/pristine col-Si full-cell (hereafter PL0-FC). To better understand this difference, a three-electrode Swagelok cell (Fig. 2d) with Li metal as a reference electrode was assembled to monitor the individual voltage profiles of the composite cathode and anode. The cathode and anode potentials of PL0-FC before charging, as depicted in Fig. 2e, are measured at 3.47 V and 3.20 V respectively. Similarly, the cathode potential for PL150-FC (Fig. 2f) is recorded at 3.51 V while the anode potential is found to be only 0.61 V, resulting in OCVs of 0.27 V and 2.90 V for PL0-FC and PL150-FC respectively. These OCV values align with the previously discussed results for coin cells. The charge curves of the cathodes in PL0-FC and PL150-FC (Fig. S16) reveal similarities, both showing three charge plateaus at 3.53 V, 4.00 V and 4.10 V, corresponding to the Fe^3+^/Fe^2+^, Li_0.5_Mn_2_O_4_/LiMn_2_O_4_ and MnO_2_/Li_0.5_Mn_2_O_4_ redox couples, respectively [46,47]. Furthermore, the charge curve around 4.10 V is also associated with the Mn^3+^/Mn^2+^ redox pairs of LMFP materials [46]. At a discharge cut-off voltage of 2.50 V, the potential of pristine col-Si increases to 1.45 V while the cathode potential decreases to 3.95 V. The absence of the discharge plateau corresponding to the Fe^3+^/Fe^2+^ redox couple (Fig. 2e and Fig. S16) indicates that the pristine col-Si fails to further lithiate the cathode to 3.20 V (Fig. 2f). In contrast, PL150-FC exhibits a complete discharge profile, demonstrating its ability to fully return active lithium for cathode lithiation. Additionally, the charge-discharge profiles (Fig. S17) of the coin cells reveal that while the Fe^3+^/Fe^2+^ plateau no longer exists during the second and third cycles in PL0-FC, it persists in PL150-FC. This confirms that prelithiation of col-Si can compensate for lithium loss in the initial cycle and ensure maximum preservation of active lithium in subsequent cycles, thereby enhancing the energy density of LIBs.

**Figure 3.**
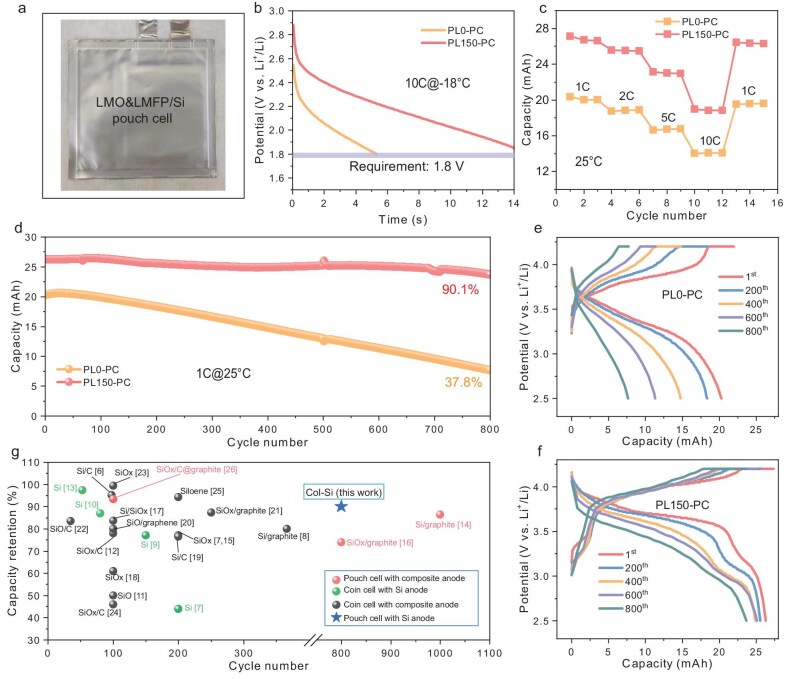
Electrochemical performance of the LMO&LMFP/col-Si pouch cell (PL0-PC) and LMO&LMFP/PL150-Si pouch cell (PL150-PC). (a) Optical image of the fabricated pouch cell with 27 mAh (1.05 mAh cm^−2^). Cold-cranking capability with (b) a current rate of 10C at −18°C, (c) rate performance at 25°C and (d) cycling performance over 800 cycles at 1C at 25°C, of PL0-PC and PL150-PC. Charge-discharge profiles per 200 cycles for (e) PL0-PC and (f) PL150-PC. (g) Cycle performance comparison between PL150-PC and other published studies. Note that corresponding references are given in Table S1 in the supplementary data.

The practical application potential of PL150-Si was assessed by constructing single-layer LMO&LMFP/PL150-Si pouch cells (referred to as PL150-PC) and LMO&LMFP/pristine col-Si pouch cells (referred to as PL0-PC). Initially, the pouch cells were charged up to an 80% state of charge (SOC), followed by a resting period of 6 hours at −18°C, and then subjected to cranking at a current rate of 10 C. This evaluation aimed to assess their cold-cranking capability, which is a critical metric for high-power batteries. Figure 3b illustrates that both PL0-PC and PL150-PC can be successfully cranked in −18°C conditions. Notably, PL150-PC demonstrates an impressive cold-cranking voltage of 2.40 V at a pulse duration of 2 s, significantly higher than that of PL0-PC (2.05 V). Furthermore, it is worth highlighting that the voltage drop in PL150-PC occurs gradually over a span of 15 s before reaching 1.80 V, whereas in contrast, there is a rapid drop to 1.80 V after only 5.3 s for PL0-PC. These findings emphasize the significant enhancement in cold start performance achieved through the prelithiation strategy employed in these batteries, effectively meeting the automotive cold-cranking requirements (at least 1.80 V at cell unit level). The rate capabilities of PL0-PC and PL150-PC were assessed at various discharge rates, as depicted in Fig. 3c. At 1 C, 2 C, 5 C and 10 C, PL0-PC demonstrates average capacities of 20.1, 18.8, 16.7 and 14.1 mAh respectively. In contrast, PL150-PC exhibits reversible capacities of 26.8, 25.5, 23.0 and 18.9 mAh respectively, revealing its superior rate performance. This significant enhancement in cold-cranking ability and overall rate performance resulting from the improved capacity through anode prelithiation ensures sufficient power output for effective engine operation under demanding conditions.

The long-cycle performance of the pouch cells at a current rate of 1 C within a voltage range of 2.50–4.20 V at 25°C is illustrated in Fig. 3d. It is worth noting that PL150-PC demonstrates an impressive initial capacity of 26.3 mAh after formation, compared to 20.3 mAh for PL0-PC, highlighting the effectiveness of prelithiation of col-Si in compensating for the initial capacity loss and providing sufficient energy density in subsequent cycles. Furthermore, the prelithiation strategy significantly enhances the long-term cycling stability of the cells as evidenced by PL150-PC retaining a high capacity of 23.7 mAh with an ultrahigh capacity retention of 90.1% after 800 cycles, whereas PL0-PC experiences severe capacity decay with only 7.7 mAh remaining and a low capacity retention of 37.8%. Detailed analysis reveals that pouch cells incorporating PL150-Si can effectively mitigate voltage polarization and delay battery capacity decline, as demonstrated by discharge medium voltage (Fig. S18) and voltage profiles (Fig. 3e and f) during cycling experiments. These results indicate that the prelithiation strategy not only increases reversible battery capacity but also improves electrode interface stability, which will be further explored in subsequent sections. The cycle performance comparison between PL150-PC and other published studies can be found in Fig. 3g and Table S1. Notably, there is limited existing literature on prelithiating pure Si anodes specifically for their applications in pouch cells, with significant commercial potential; however, our study demonstrates that pouch cells with prelithiated col-Si anodes exhibit significantly increased reversible capacity and prolonged cycle lifespan, representing a significant breakthrough towards large-scale adoption of Si anodes. Importantly, our direct immersion prelithiation approach using Li-Naph solutions offers a scalable and industry-compatible process, paving the way for seamless integration into existing manufacturing lines, as illustrated in Fig. S19.

The pouch cells utilize a semi-solid-state GBL-based electrolyte, which is widely recognized for its enhanced safety characteristics. Consequently, the assembled cells undergo rigorous tolerance testing under extreme conditions. As shown in Fig. S20, a PL150-PC cell at a 100% SOC after 800 cycles was folded, horizontally cut and subjected to direct combustion tests. Notably, the PL150-PC continued to power a blue LED light without ignition or explosion, demonstrating the inherent safety benefits of the semi-solid electrolyte system. These findings underscore the potential of semi-solid-state electrolyte systems for improving battery safety, which is particularly significant for large-scale applications.

### Effect of prelithiation on the stability of electrode/electrolyte interphase

The interfacial morphology and properties of col-Si and PL150-Si were examined before and after three cycles using SEM, TEM and atomic force microscopy (AFM). As depicted in Fig. 1g, the pristine col-Si exhibits a remarkably smooth surface. After three cycles, the surface of col-Si displays fewer electrolyte decomposition products compared to PL150-Si (Fig. S21). TEM analysis confirms that PL150-Si forms a thicker and more uniform SEI layer on its surface when compared to col-Si. Additional investigation through AFM reveals that the surface of PL150-Si exhibits increased flatness along with decreased Young's modulus, suggesting enhanced elasticity within the SEI layer. These experimental findings provide evidence that prelithiated col-Si possesses a thicker and more uniform SEI layer with improved elasticity, serving as an effective buffer layer for mitigating stress induced by substantial volumetric changes in col-Si particles during cycling.

**Figure 4.**
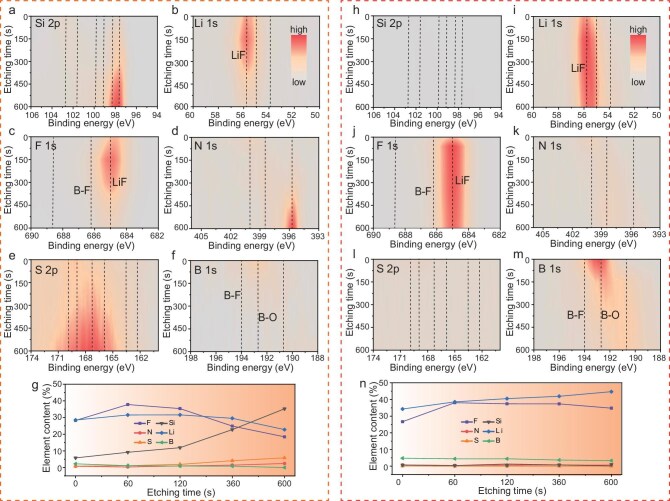
Chemical composition and distribution of SEI. Contour maps of Si 2p, Li 1s, F 1s, N 1s, S 2p and B 1s XPS patterns and the element content with Ar^+^ sputtering of (a–g) the cycled col-Si and (h–n) cycled PL150-Si anode. The anodes were harvested from PL0-FC and PL150-FC after three cycles. Note that the original XPS patterns are provided in Figs S24 and S25.

Subsequently, in-depth XPS measurements were employed to further investigate the differences in interfacial chemical composition between the two cycled anodes. From Fig. 4 and Fig. S24, it is evident that the col-Si anode after cycling exhibits Si signals corresponding to the Li_x_Si species [48], which becomes increasingly prominent with etching. In sharp contrast, the Si signals in PL150-Si are very weak, even after 600 s of sputtering. In addition, it can also be found from the Li 1s spectrum that the content of Li compounds from the decomposition of the electrolyte on the surface of col-Si after cycling is significantly less than that of PL150-Si and becomes weaker after 360 seconds of sputtering. These observations strongly indicate that prelithiation contributes to a thicker SEI layer on the PL150-Si surface, aligning well with the previous SEM and TEM results. The analysis of F 1s spectra and elemental composition distribution (Fig. S24 and Fig. 4) demonstrates a significantly higher F content on the surface of PL150-Si compared to col-Si. This observation suggests an enhanced decomposition of lithium salts, thereby promoting the formation of an F-rich (particularly LiF) interphase. Furthermore, LiF in the SEI covering the PL150-Si interface exhibits a more uniform distribution in the depth direction. Several studies have highlighted that a SEI layer enriched with LiF can effectively accommodate the deformation of the Li-Si alloy, mitigate active lithium depletion and significantly enhance the cyclic stability of the Si anode [31,45,49]. Therefore, the SEI film that covers the PL150-Si surface, which is rich in LiF and uniformly distributed, is more conducive to maintaining the interphasial stability of the Si particles during cycling. By comparing the N 1s, S 2p and B 1s spectra results (Fig. 4d–f and k–m, and Fig. S25), it can be inferred that the surface of PL150-Si contains a higher content of B compounds, but a lower concentration of S and N compounds compared to col-Si. This suggests that there is greater decomposition of BF_4_^−^ on the surface of PL150-Si, while col-Si experiences more decomposition of TFSI^−^. Additionally, it can be observed that the decomposition products of TFSI^−^ are primarily located in the inner layer of the SEI film, appearing after a sputtering time of ∼300 seconds, while the decomposition products of BF_4_^−^ are predominantly distributed in the outer layer of the SEI film. It has been demonstrated that the SEI film constructed with B compounds helps reduce electrode polarization, enhancing the rate performance and low-temperature discharge capability of the battery. This explains why the PL150-Si system exhibits lower electrode polarization and superior cold-start capability compared to the col-Si system presented in Fig. 3.

Subsequently, density functional theory (DFT) calculations were employed to elucidate the underlying reasons for the substantial alterations in the electrolyte decomposition behavior at the Si surface induced by prelithiation treatment. It can be found from Fig. S26 that the reductive activity of each component in the electrolyte follows the sequence of LiTFSI > FEC > GBL > LiBF_4_. During the charging process (Fig. S16b), the potential of the pristine col-Si anode gradually decreases from an OCP of 3.20 V. During the initial charging, LiTFSI with the highest reduction activity will initially accept electrons to undergo reduction decomposition, thereby playing a pivotal role in the formation of the inner layer structure for SEI film. As the voltage decreases, the FEC gradually decomposes, resulting in an additional accumulation of LiF in the outer SEI. Such SEI constructed by the decomposition of FEC and LiTFSI may inhibit the decomposition of GBL and LiBF_4_ with lower reduction activity. Consequently, the SEI of the cycled col-Si anode comprises lower B-containing compounds and is mainly located in the outermost layer. The OCP of the PL150-Si anode, in contrast, is as low as 0.61 V, which may result in electrolyte reduction decomposition when it comes into contact with the electrolyte, including exhibiting the lowest reduction activity of LiBF_4_. When multiple electrolyte components undergo simultaneous reduction and decomposition, the resulting SEI film exhibits a more uniform distribution in terms of depth, as presented in Fig. 4n. Hence, the disparities in SEI film composition and properties after cycling between prelithiated and non-prelithiated Si electrodes primarily stem from the distinct OCP of the two electrode-assembled batteries. The former exhibits a lower OCP, leading to simultaneous reduction decomposition of electrolyte components and resulting in an SEI film with more uniform composition distribution and a higher content of LiF and B-class compounds, thereby imparting greater structural stability and reduced polarization characteristics to the SEI film.

**Figure 5.**
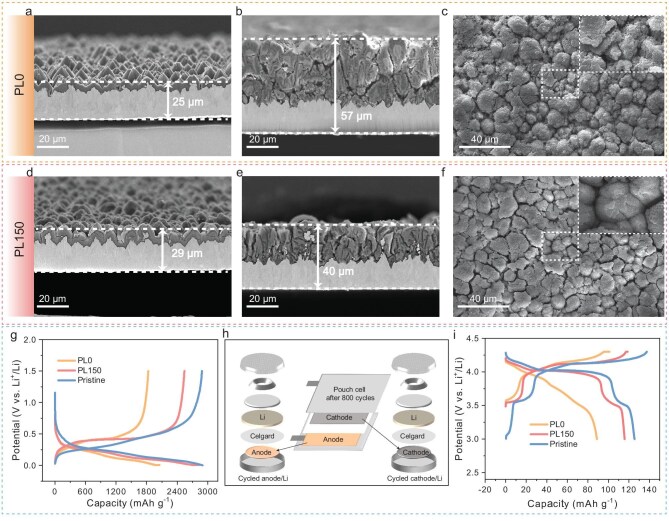
Morphological evolution and performance of electrodes after 800 cycles in pouch cells. Cross-sectional SEM images of pristine (a) col-Si, (d) PL150-Si and anodes after 800 cycles in (b) PL0-PC and (e) PL150-PC. Top-view SEM images of col-Si anodes after 800 cycles in (c) PL0-PC and (f) PL150-PC. Cycle performance of half-cells with pristine electrodes and cycled electrodes retrieved from PL0-PC and PL150-PC after 800 cycles: (g) anode/Li cells, (i) cathode/Li cells and (h) a diagram of pouch-cell disassembly and coin-cell assembly.

The SEM images in Fig. 5 depict the Si anodes after 800 cycles. It is apparent that the thickness of the col-Si electrode increased from 25 μm to 57 μm, while the thickness of PL150-Si only increased by 11 μm after cycling. Upon observing the morphology of Si particles, it is evident that due to severe volume expansion/contraction, col-Si almost completely shattered after cycling, while PL150-Si still maintained intact particle morphology. This outcome further validates the exceptional structural stability of the SEI film formed after prelithiation.

To investigate the capacity recovery of the cathode and anode after extensive cycling, the pouch cell was disassembled after 800 cycles. The cathode and anode were trimmed and reassembled with fresh lithium foil to form half-cells (Fig. 5h). As shown in Fig. 5g, the capacity of the PL150-Si electrode from the PL150-PC reaches 2542 mAh g^−1^, which is very close to that of a fresh Si anode. In contrast, the capacity recovery of col-Si from the PL0-PC only reaches 1803 mAh g^−1^, demonstrating the significantly superior stability of PL150-Si compared to col-Si. Notably, the capacity recovery of the cathode (Fig. 5i) from the PL150-PC is also significantly higher than that from the PL0-PC, recovering to 116 mAh g^−1^, whereas the PL0 system only reaches 89 mAh g^−1^. Examination of the corresponding charge-discharge curves reveals that the low capacity of the cathode from the PL0-PC mainly stems from the disappearance of the Mn^3+^/Mn^2+^ redox platform. This is likely due to the formation of the SEI layer on the col-Si anode during the initial charge in the PL0-PC cell, which consumes a substantial amount of active lithium. As a result, some of the cathode material remains in a high-voltage delithiated state, which can induce Mn dissolution and ultimately cause the disappearance of the corresponding plateau. In summary, the use of prelithiated Si anodes not only promotes the formation of a uniform and durable SEI layer on Si anode surfaces, enhancing cycling stability, but also strengthens the cathode material structure during extended cycling processes. As a result, this significantly improves the long-term cycle stability of the pouch cells.

## DISCUSSION

This study found that the feasibility of prelithiating silicon anodes using the traditional Li-Naph reagent mainly depends on the structural state of the silicon material. Specifically, the initial lithiation potential of crystalline silicon is significantly lower than the reduction potential of the Li-Naph reagent, while the initial lithiation potential of amorphous silicon is higher, allowing lithium ions and electrons to migrate from the Li-Naph reagent to the amorphous silicon anode, achieving efficient prelithiation. By simply adjusting the immersion time of the silicon anode in the prelithiation reagent, the degree of prelithiation can be precisely controlled.

A full-cell, composed of a LiMn_2_O_4_ and LiMn_0.7_Fe_0.3_PO_4_ composite cathode and a silicon anode prelithiated for 150 minutes, shows a significant increase in ICE from 74.8% to 97.2%. More importantly, the related high-safety pouch cell with semi-solid-state electrolytes exhibits excellent long-cycle stability, rate performance and low-temperature cold-start capability. This is mainly due to the improved capacity achieved by anode prelithiation, which ensures sufficient power output for effective engine operation in demanding conditions. Additionally, the reduction in the silicon anode potential after prelithiation induces simultaneous reductive decomposition of electrolyte components upon contact with the anode. This process forms a robust, compositionally uniform and low-impedance SEI film, significantly enhancing the long-cycle stability and rate performance of the silicon anode. Furthermore, the efficient utilization of active lithium ions allows for the full lithiation of the cathode material during discharge, lowering the proportion of delithiated material, inhibiting the dissolution of transition metal ions and mitigating the capacity decay of the cathode material. This study demonstrates the successful prelithiation of pure silicon anodes using traditional, low-cost and environmentally friendly prelithiation reagents, and achieves long-cycle stability in pouch cells. This advancement marks an important step towards the large-scale practical application of silicon anodes and provides valuable guidance for designing high-energy-density and high-power-density LIBs.

## Supplementary Material

nwaf084_Supplemental_File
