# Supplementary material for: Integrated prelithiation and SEI engineering for high-performance silicon anodes in lithium-ion batteries
Source: Natl Sci Rev. 2025 Mar 3;12(7):nwaf084. doi: 10.1093/nsr/nwaf084 (PMC12168764; doi:10.1093/nsr/nwaf084)
Supplement: nwaf084_Supplemental_File [file nwaf084_supplemental_file.pdf]

Integrated Prelithiation and SEI Engineering for High-Performance Silicon Anodes in Lithium-Ion Batteries

Lijiao Quan[a,b,#], Qili Su[c,#], Haozhe Lei[a], Wenguang Zhang[a,b], Yingkang Deng[a,b], Jiarong He[a,b], Yong Lu[c], Zhe Li[c,*], Haijing Liu[c,*], Lidan Xing[a,b,*] and Weishan Li [a,b]

[a]School of Chemistry, South China Normal University, Guangzhou 510006, China;

[b]National and Local Joint Engineering Research Center of MPTES in High Energy and Safety LIBs, Engineering Research Center of MTEES (Ministry of Education), Research Center of BMET (Guangdong Province), and Key Lab. of ETESPG(GHEI), South China Normal University, Guangzhou 510006, China;

[c]Battery Research and Development, Battery Propulsion & Sustainability, General Motors, Shanghai 201206, China

Experimental section

Preparation of gel polymer electrolyte

Lithium bis(trifluoromethane sulfonyl) imide (LiTFSI, ≥99%), lithium tetrafluoroborate ($LiBF_4$, ≥99%) and Dimethyl carbonate (DMC) were purchased from DoDoChem. Fluoroethylene carbonate (FEC) was provided from Guangzhou Tinci Materials Technology Co., Ltd., China and gamma-butyrolactone (GBL, ≥98%) was provided by Zhangjiagang Guotai-Huarong New Chemical Materials Co. Ltd., China. FEC and GBL were dried over molecular sieves (4 Å, 3-5 mm) for 48 h prior to use. The liquid electrolyte was prepared by adding 0.8 M LiTFSI and 0.8 M $LiBF_4$ to a mixed solvent of FEC and GBL (3:7 in weight), in which FEC is recognized as an effective component to improve the stability of the silicon electrode/electrolyte interphase [1]. The gel polymer electrolyte (GPE) was obtained by mixing 205 wt% of a PVDF-HFP solution (5 wt% PVDF-HFP dissolved in 200 wt% DMC) with 95 wt% of a liquid electrolyte, as described in previous works [2,3]. Specifically, GPE could be in-situ formed by placing the gel precursor solution on the surface of electrodes in an argon-filled glove box for 1.5 hours to evaporate DMC solvent.

Preparation of electrodes and prelithiation of silicon anode

The slurry of composite cathode was synthesized by blending $LiMn_2O_4$(LMO, 44 wt%), $LiMn_{0.7}Fe_{0.3}PO_4$(LMFP, 44 wt%), $Li_{1.3}Al_{0.3}Ti_{1.7}(PO_4)_3$ solid electrolyte (LATP, 5 wt%, aiming to improve the battery rate capability), super P (2.9 wt%), single-walled carbon nanotube (0.1 wt%) and poly(vinylidene difluoride) (PVDF, 4 wt%) in N-methyl-2-pyrrolidinone (NMP) solvent. Similarly, two commercial silicon anodes were fabricated in the same manner as the composite cathode, and the weight ratio of silicon powder (nano-sized silicon or micron-sized silicon),

super P and carboxymethyl cellulose sodium (CMCNa) was controlled at 6:2:2, where CMCNa was firstly dissolved in deionized water. After stirring, the resultant homogeneous slurries were casted onto a carbon-coated aluminum foil for the composite cathode and a copper foil for the silicon anodes, followed by vacuum drying at 120 ℃ for overnight to completely evaporate the NMP or water. Finally, the obtained electrodes were punched into circles with a diameter of 12 mm for all the cathode, the nano-sized anode and the micron-sized anode, and the average mass loading were about 9.78, 1.33, and 0.53 mg cm^{-2}, respectively. The columnar silicon film (col-Si) electrode was fabricated in a roll-to-roll process via physical vapour deposition (PVD). Specifically, silicon was deposited onto the copper foil at a constant deposition rate by sputtering a pure Si target in Ar gas at a flow rate of 2 standard cubic centimeters per minute. The thickness of the col-Si can be determined by controlling the deposition time. The col-Si anode exhibits particle sizes ranging from 6.6 to 12 μm, with a deposition thickness of approximately 7 μm and a mass loading of 0.98 mg cm^{-2}. It should be noted that the copper foil with a thickness of 18 μm, characterized by a roughened layer, allows the formation of columnar structures and ensures the adhesion of the silicon.

The prelithiation reagent, designated as Li-Naph solution, was prepared by dissolving lithium metal (battery grade) in a 0.5 M solution of naphthalene (battery grade) in dimethylether (DME, 99%, Aladdin) under a magnetic stirring at room temperature for 3 hours. Li : naphthalene molar ratio was set at 1.2 : 1 to ensure sufficient Li for the preparation of the prelithiation reagent. The prelithiation process was conducted simply by immersing the punched Si anode in 1 mL Li-Naph solution at a certain time at room temperature. After reacting for the designed time, the prelithiated Si anode was rinsed with DME several times and dried in vacuum chamber for 60 min. The preparation of Li-biphenyl (Li-BP) reagent and its prelithiation process

for silicon anode are identical to those for Li-Naph reagent. All of the above prelithiation operations were performed in an argon-filled glove box.

Electrochemical measurements

CR2025 Si/Li coin cells were assembled with Celgard 3501 separator (25 μm in thickness) and 60 μL of the liquid electrolyte, then discharged to 0.005 V and recharged to 1.5 V at a current rate of 0.05 C (1 C = 3600 mAh g^{-1}) for three cycles. The diameter and thickness of lithium foil for coins cell are 15.6 mm and 450 μm, respectively. To fabricate GPE-introduced coin cells, a gel precursor solution was initially applied onto the surfaces of a composite cathode (12 mm), a pristine or prelithiated Si anode (12.5 mm), and a separator (19 mm). This separator consists of a polyethylene (PE) membrane (9 μm thick) with a 3-μm-thick LATP coating on one side. Subsequently, the composite cathode, the LATP-coated PE separator, and the Si anode—each containing the GPE—were stacked to assemble CR2025-type full cells with an LMO&LMFP/LATP-coated PE/Si configuration. Notably, due to the poor reduction stability of LATP, the LATP-coated side of the separator is placed in contact with the gelated cathode, while the uncoated PE side interfaces with the gelated anode.

A Swagelok cell comprising three electrodes was fabricated using the GPE-introduced composite cathode as the working electrode, the GPE-introduced pristine or prelithiated Si anode as the counter electrode, and the lithium metal as the reference electrode. The coin full-cells and the Swagelok cell underwent charging to 4.2 V at a constant current rate of 0.05 C (1 C = 122 mAh g^{-1}), followed by discharging to 2.5 V at 0.2 C for the initial three cycle. The Land Test System (CT2001A, Wuhan, China) was employed for testing coin cells, while the Swagelok cell underwent testing on the electrochemical station of EC-Lab (PGSTAT302N). For pouch cells, the LMO&LMFP composite cathode and the pristine or prelithiated Si anode were meticulously

cut into rectangles with dimension of 50*55 mm and 52*57 mm, respectively. The amount of electrolyte added onto the electrodes is 1000 μL. Subsequently, a layer of GPE was in-situ formed on their surfaces utilizing a process akin to that employed for coin cells. After that, the GPE-introduced composite cathode and Si anode were sequentially stacked and enclosed within a laminated aluminum pouch. To fully activate the electrodes, pouch cells with a compressive pressure of 30 psi were initially charged to 4.2 V at 0.05 C and discharged to 2.5 V at 0.2 C for the first cycle, followed by the three charge-discharge cycles at 1C. After activation, pouch cells with 80% SOC were placed at -18 ℃ for 6 hours, and then cranked at a current rate of 10 C. Subsequent to the aforementioned procedures, pouch cells underwent charging at a constant current rate of 1 C up to 4.2 V, followed by a period of constant voltage at 4.2 V with a current limit of 0.05 C. Subsequently, discharge occurred at incremental current densities of 1 C, 2 C, 5 C, 10 C, and back to 1 C, each for a duration of 3 cycles per rate. For the assessment of cycling performance, pouch cells were charged at a constant current rate of 1 C to 4.2 V, maintained at a fixed voltage of 4.2 V with a current limit of 0.05 C, and discharged to 2.5 V with 1 C current rate at 25 ℃.

Material characterization

X-ray diffraction patterns (XRD, Rigaku Ultima IV, Japan), Raman (WITec Apyron 300R, with an excitation wavelength of 532 nm) and transmission electron microscopy (TEM, FEI-Talos F200X, America) were used to characterize the structure of different Si materials. Notably, the Raman spectra of the prelithiated Si anodes were examined in a gas-tight device to eliminate the influence of air. After cycling, the electrodes extracted from LMO&LMFP/Si full cells were rinsed several times with DMC, followed by drying in the vacuum chamber for 60 min. Finally,

the resultant electrodes were analyzed using field emission scanning electron microscope (FESEM, JSM-7900F, Japan), TEM and in-depth X-ray photoelectron spectroscopy (XPS, ESCALAB 250) to investigate their morphology and surface compositions. The electrode samples were cut using a cross-section polisher (IB19520CCP) and subjected to evaluation of their cross-sectional morphology. Note that the samples tested by XPS and SEM were prepared and transferred under the protection of an argon atmosphere to exclude air interference. The electrical conductivity and resistivity of Si anodes were measured using a four-probe resistivity tester (Hengao-RST9/Huayue-ST2722, China).

Theoretical calculations

All calculations were performed using the Gaussian 16 package. The molecular structures were optimized utilizing the B3LYP method and the 6-311++G(d, p) basis set at 298.15 K [4]. To simulate electrolyte environment, the Polarization Continuum Model (PCM) method was applied with a dielectric constant of 20.5 (acetone) [5]. Frequent analyses were also conducted employing the same basis set to ensure the optimization of each stationary point. Equation (1) was used to calculate the electron affinity energy (EAE) and to evaluate the reducing activity of the structures discussed, where E(M) and $E(M+e)^-$ are the optimized energy of the molecule before and after it gains an electron, respectively.

$$EAE = E\,(M+e)^{-} - E\,(M) \qquad (1)$$

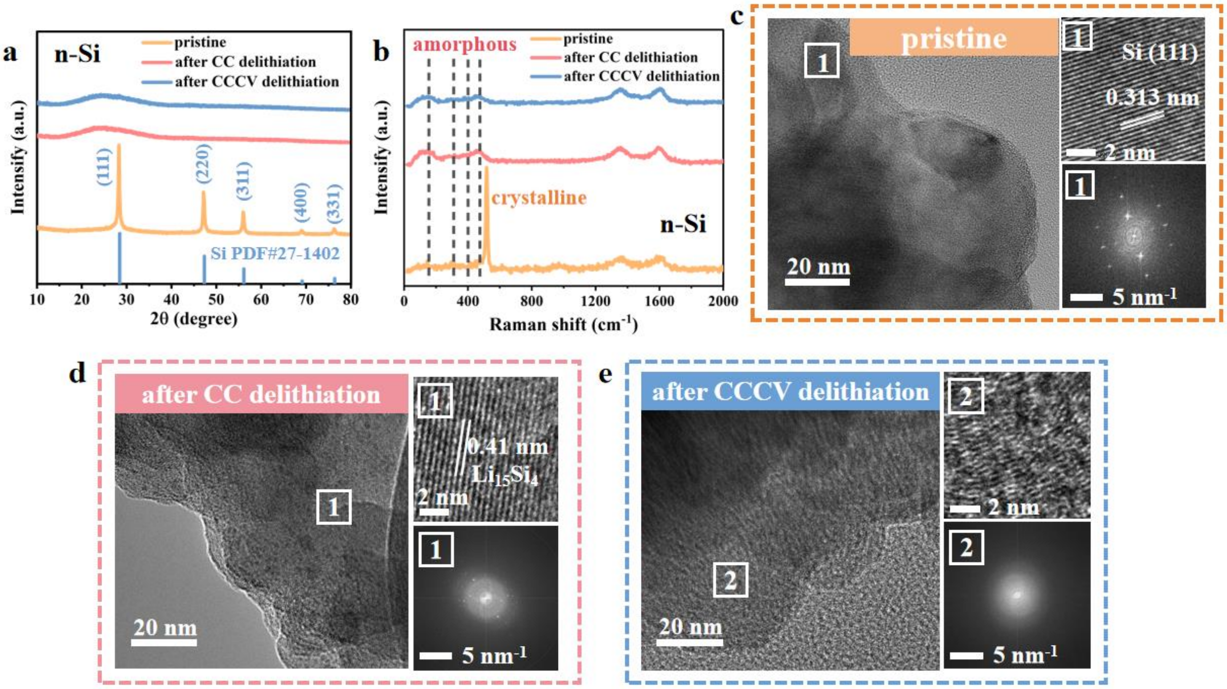

Figure S1. XRD spectra (a) and Raman spectra (b) of pristine n-Si and n-Si anodes after CC or CCCV delithiation. High-resolution TEM images and corresponding Fast Fourier Transform images of pristine n-Si (c) and n-Si electrode after CC (d) or CCCV (e) delithiation.

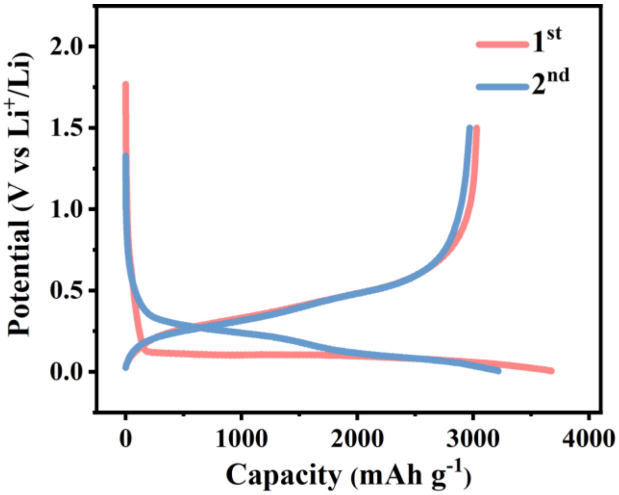

Figure S2. Voltage profiles during the initial two cycles of commercial n-Si/Li half-cells.

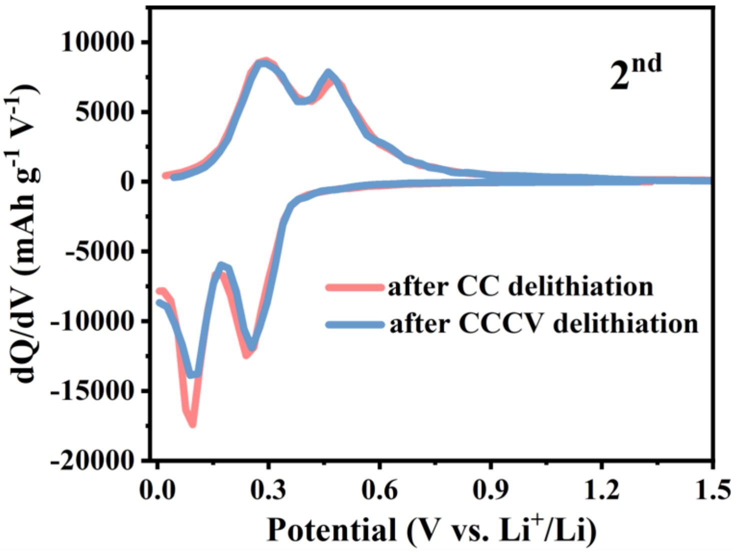

Figure S3. DQ/dV curves of the second cycle in n-Si/Li cells after CC or CCCV delithiation.

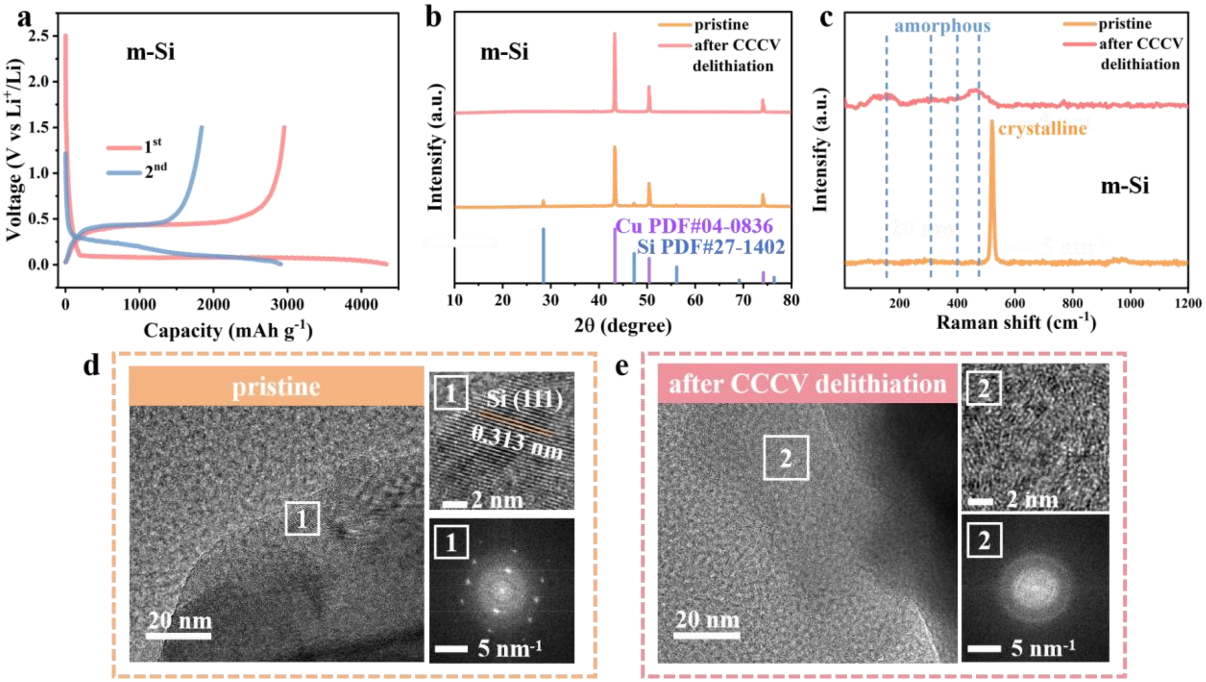

Figure S4. Voltage profiles during the initial two cycles of commercial m-Si/Li half-cells (a). XRD spectra (b) and Raman spectra (c) of pristine m-Si and m-Si anodes after CCCV delithiation. TEM images and corresponding Fast Fourier Transform images of pristine m-Si (d) and m-Si after 1st CCCV delithiation (e).

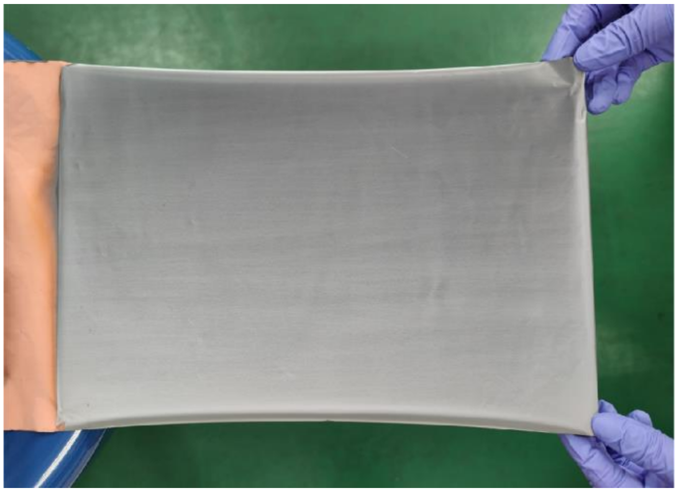

Figure S5. Optical image of the amorphous col-Si electrode prepared through physical vapor deposition (PVD) method.

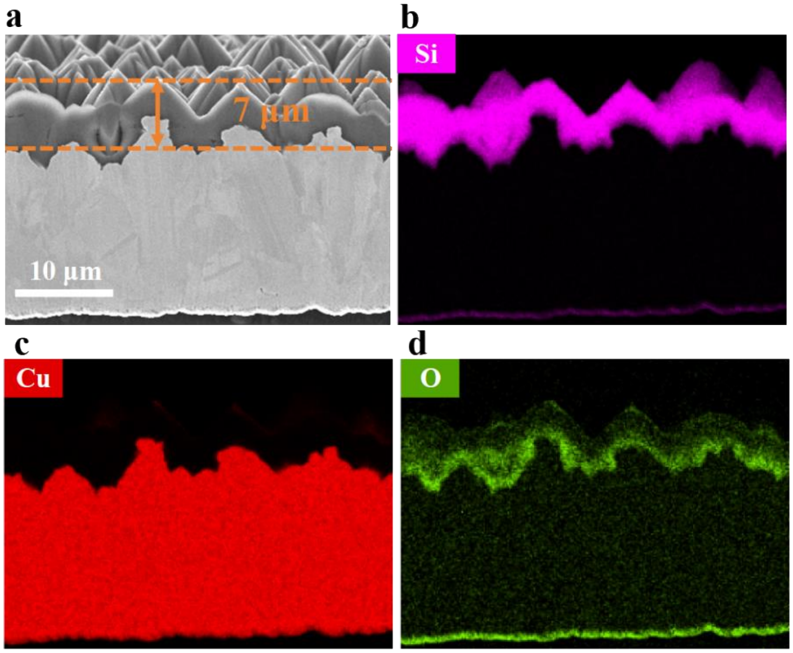

Figure S6. Cross-sectional SEM images of the col-Si electrode and corresponding Si, Cu and O element distribution.

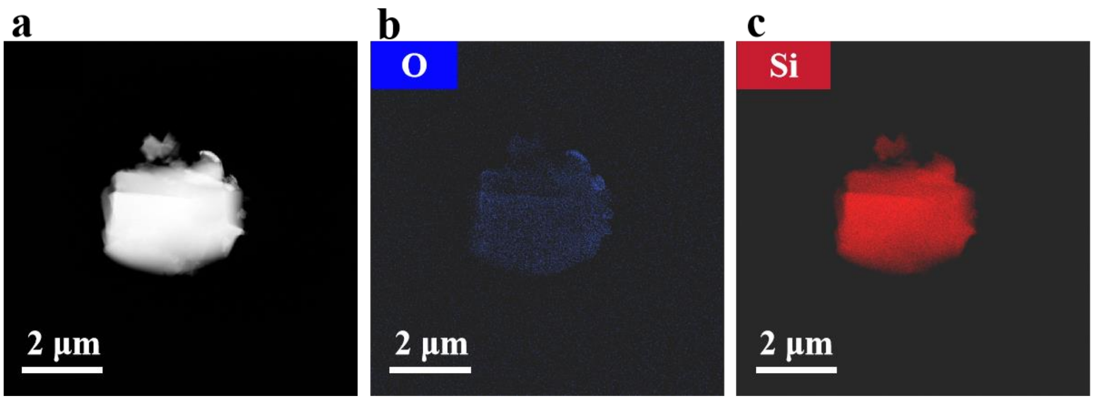

Figure S7. TEM images of col-Si particle and corresponding O and Si element distribution.

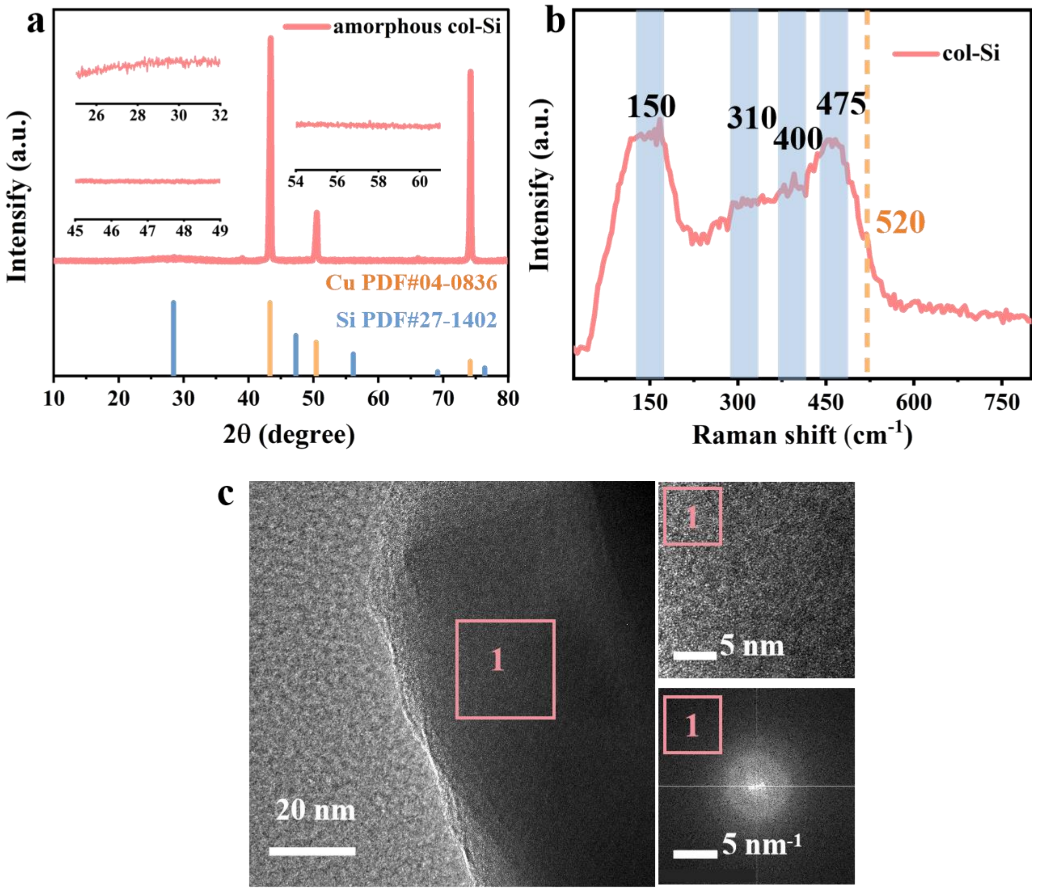

Figure S8. XRD spectra (a), Raman spectra (b), high-resolution TEM images and corresponding Fast Fourier Transform images (c) of pristine col-Si.

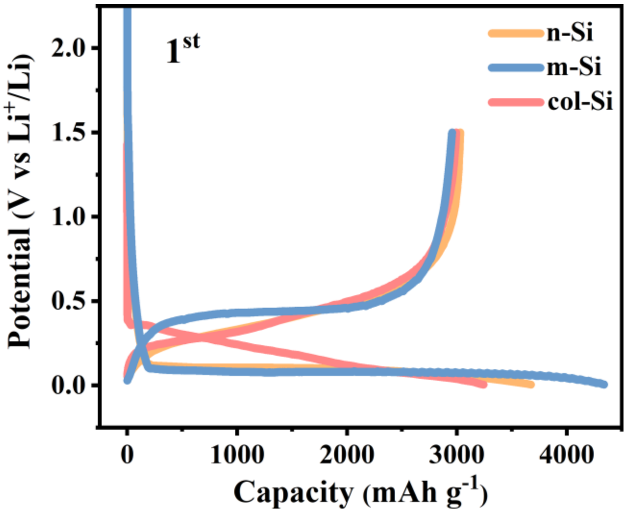

Figure S9. Initial charge-discharge profiles of n-Si/Li, m-Si/Li, and col-Si/Li half-cells.

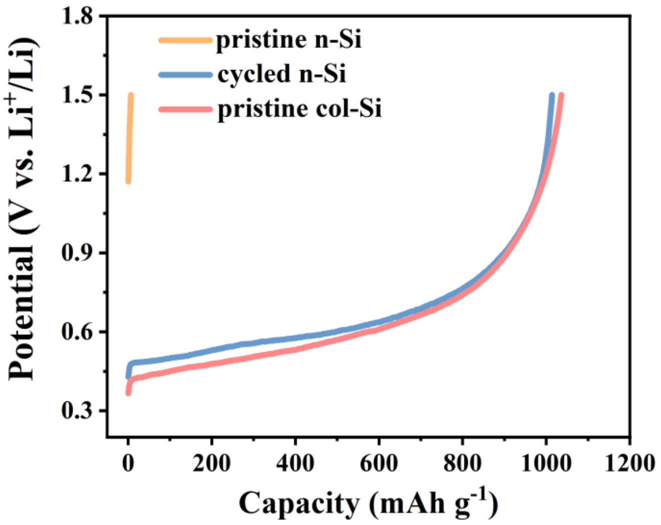

Figure S10. Delithiation process of half-cells with pristine n-Si anode, n-Si anode after one cycle, and pristine col-Si anode, all prelithiated in Li-bipheny reagent for 150 minutes.

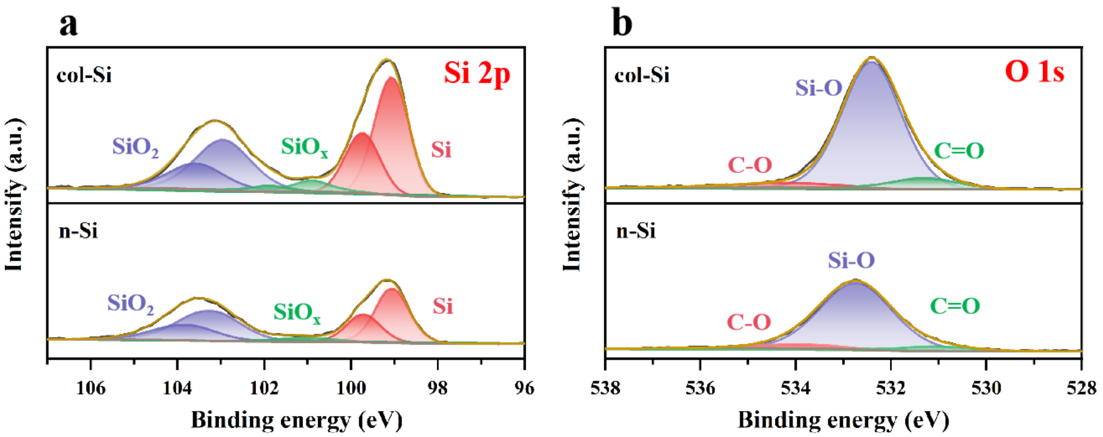

Figure S11. Comparison of Si 2p (a) and O 1s (b) XPS patterns of amorphous col-Si material and crystalline n-Si material.

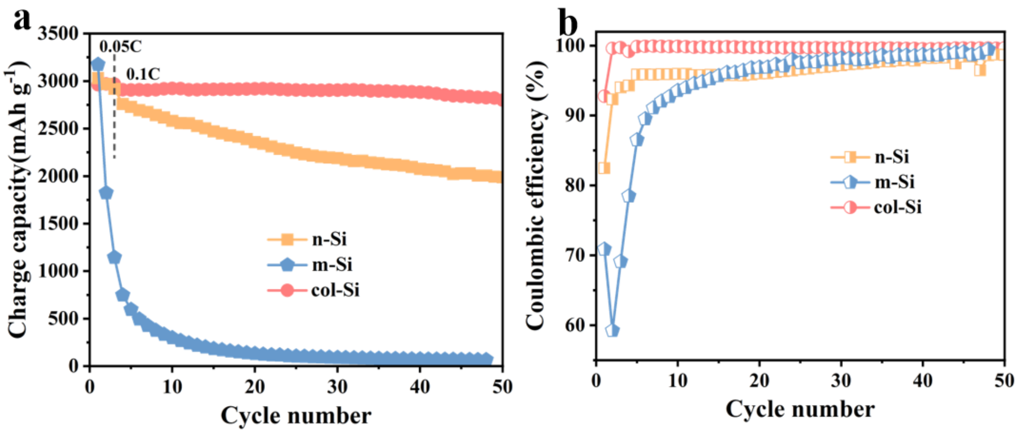

Figure S12. Comparison of cycling performance (a) and Coulombic efficiency (b) of half cells with different silicon materials.

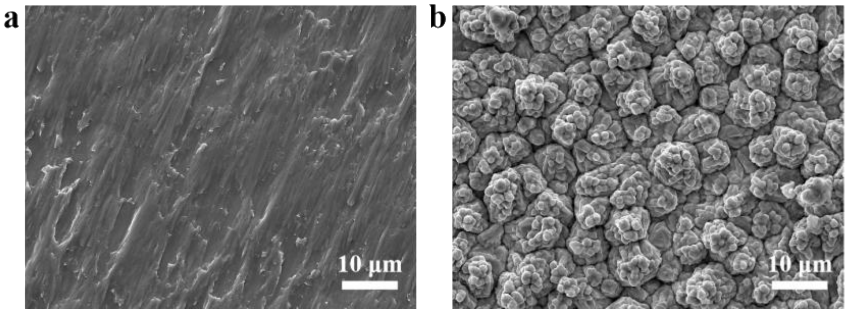

Figure S13. SEM images of the plated (a) and the roughened (b) Cu current collector.

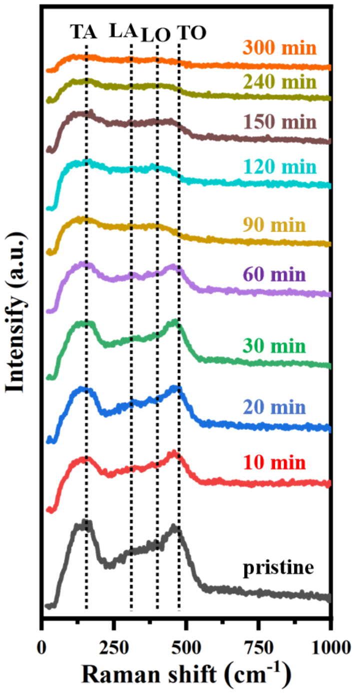

Figure S14. Raman spectra of col-Si electrodes prelithiated for different times. The four peaks represents the transverse acoustic (TA), longitudinal acoustic (LA), transverse optical (TO) and longitudinal optical (LO) modes of amorphous Si.

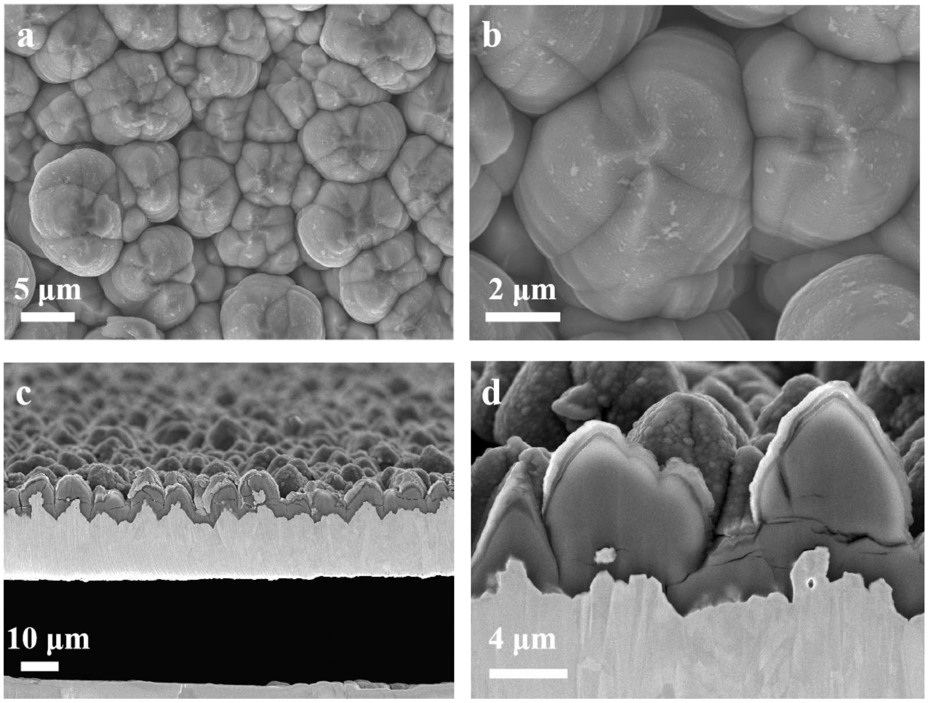

Figure S15. Top-view (a, b) and cross-sectional (c, d) SEM images of the col-Si after 150 minutes of prelithiation (PL150-Si).

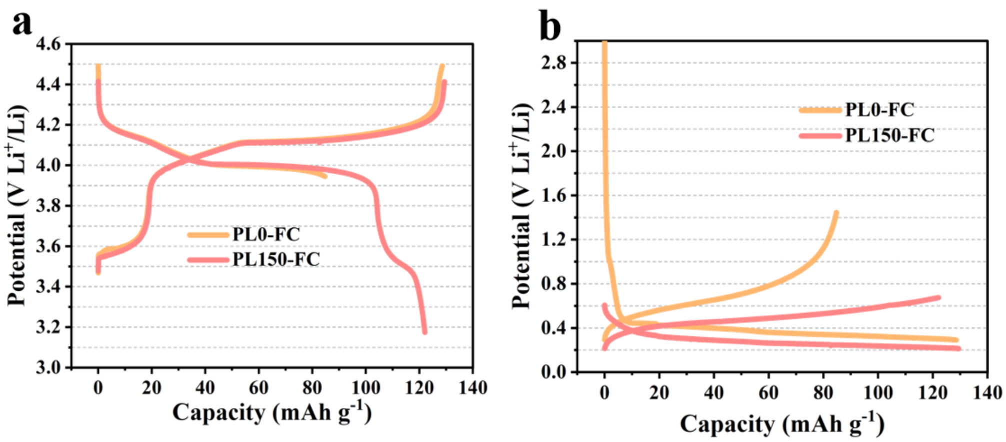

Figure S16. Comparison of separated voltage profiles of the cathodes (a) and the anodes (b) obtained from the three-electrode cells of PL0-FC and PL150-FC.

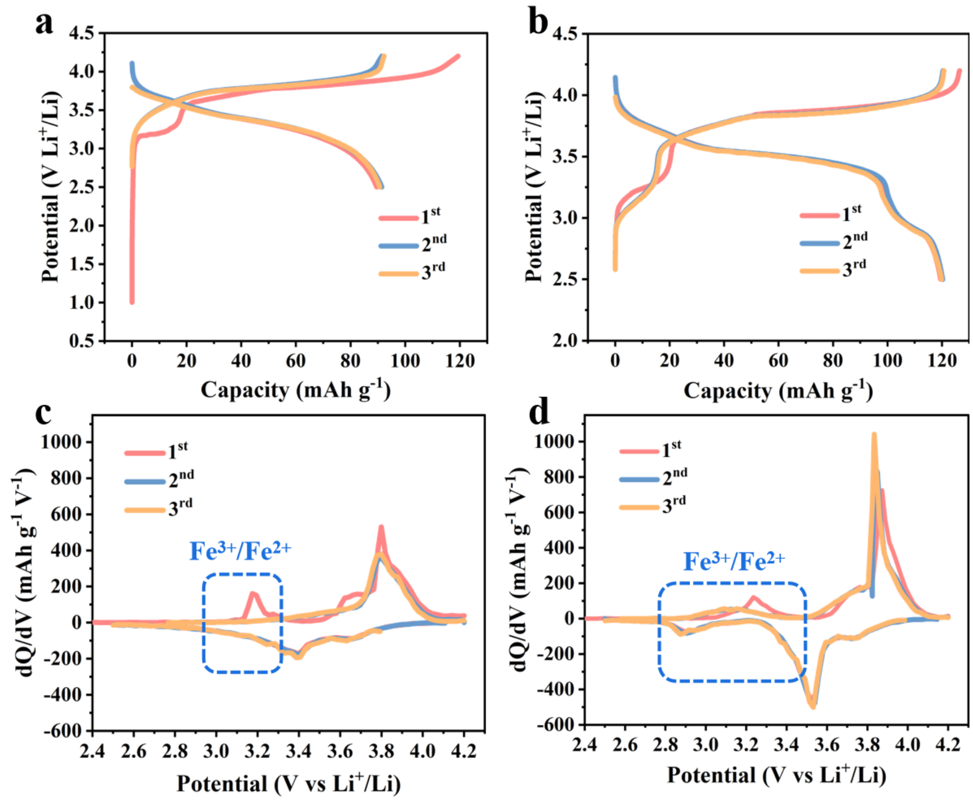

Figure S17. The first three charge-discharge profiles and corresponding dQ/dV profiles of coin cells of PL0-FC (a, c) and PL150-FC (b, d).

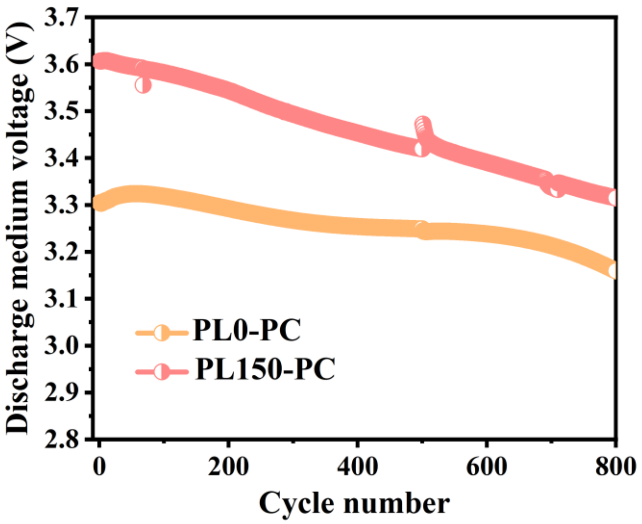

Figure S18. Discharge medium voltage of LMO&LMFP/pristine col-Si pouch cell (PL0-PC) and LMO&LMFP/PL150-Si (PL150-PC) pouch cell at 1C at 25℃ over 800 cycles.

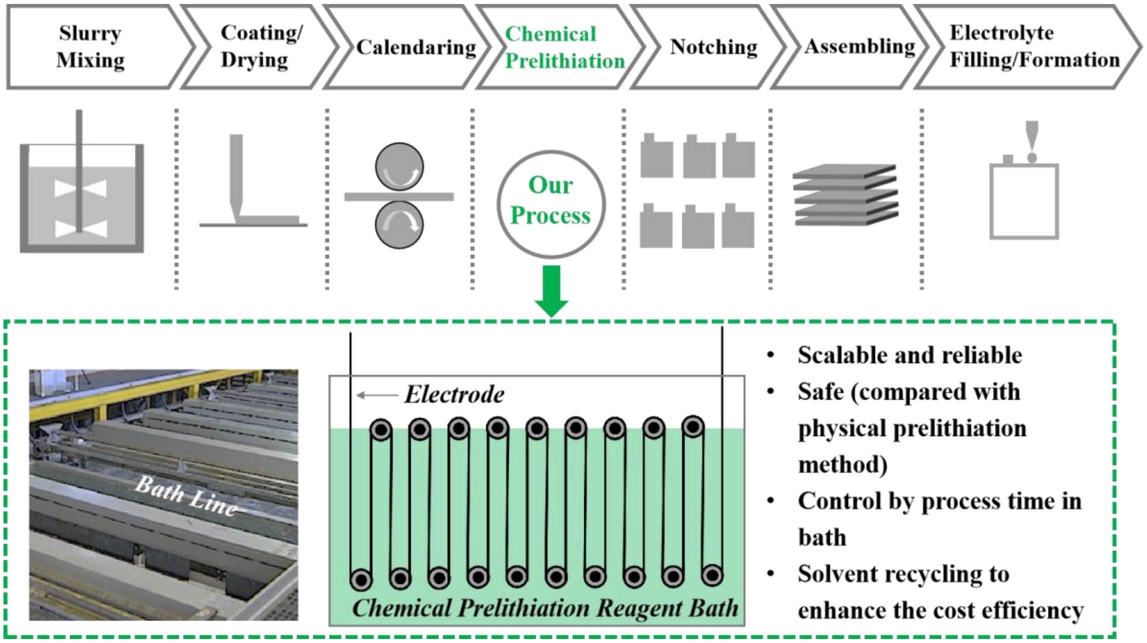

Figure S19. Schematic illustration of direct immersion prelithiation method for integration into existing production processes.

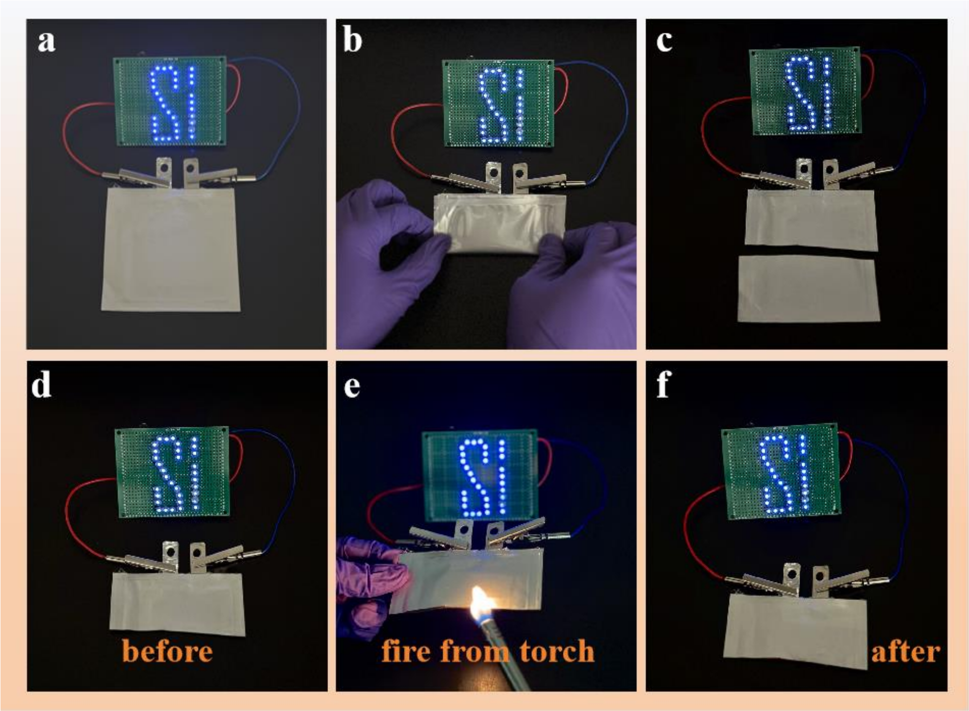

Figure S20. Tolerance testing of PL150-PC. LED powered by PL150-PC after 800 cycles (a). The blue light remains on even after horizontally folding (b), cutting (c) and burning (d-f).

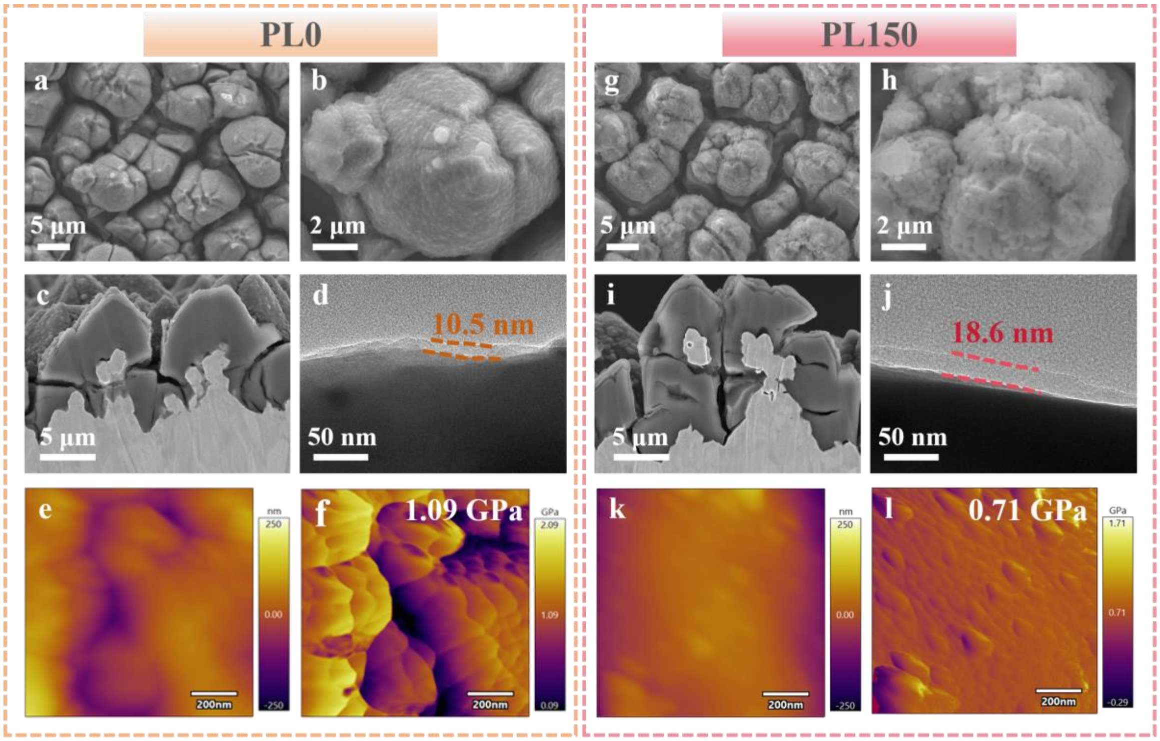

Figure S21. Surface morphology of the cycled col-Si anodes. Top-view and cross-sectional SEM images (a-c, g-i), TEM images (d, j), AFM images (e, k) and Young's modulus (f, l) of the cycled col-Si (a-f) and cycled PL150-Si (g-l). The anodes were extracted from PL0-FC and PL150-FC after 3 cycles, respectively.

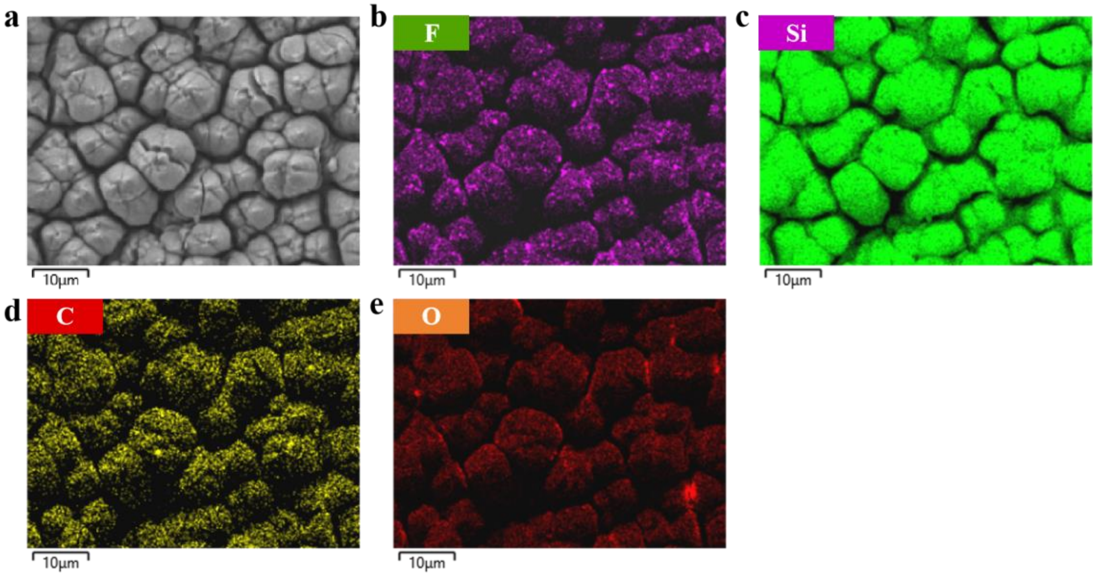

Figure S22. Top-view SEM image (a) and element mapping (b-e) of col-Si anode extracted from coin cell of PL0-FC after 3 cycles.

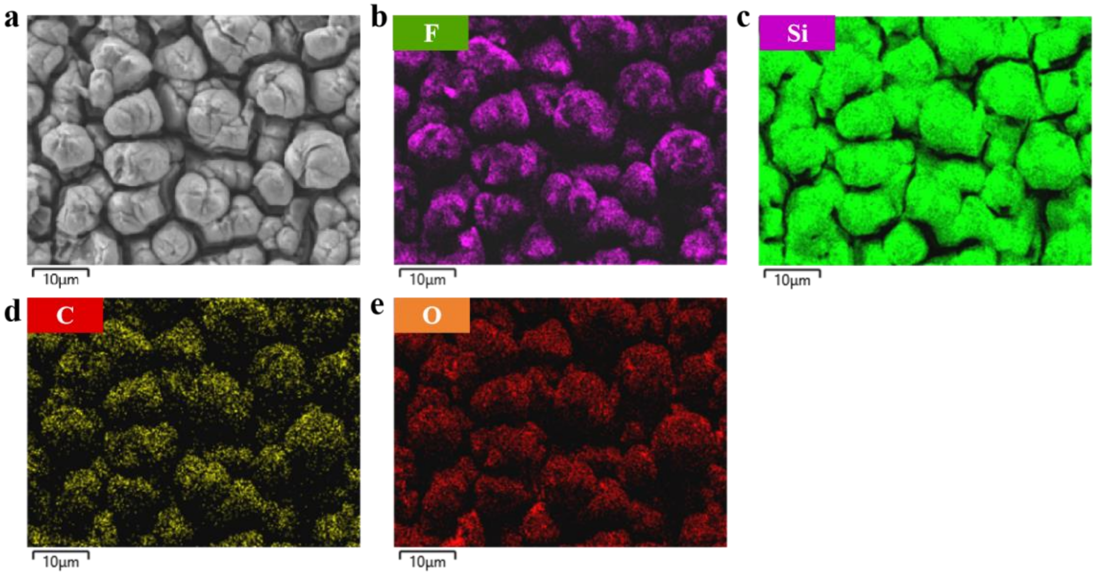

Figure S23. Top-view SEM image (a) and element mapping (b-e) of the PL150-Si anode extracted from coin cell of PL150-FC after 3 cycles.

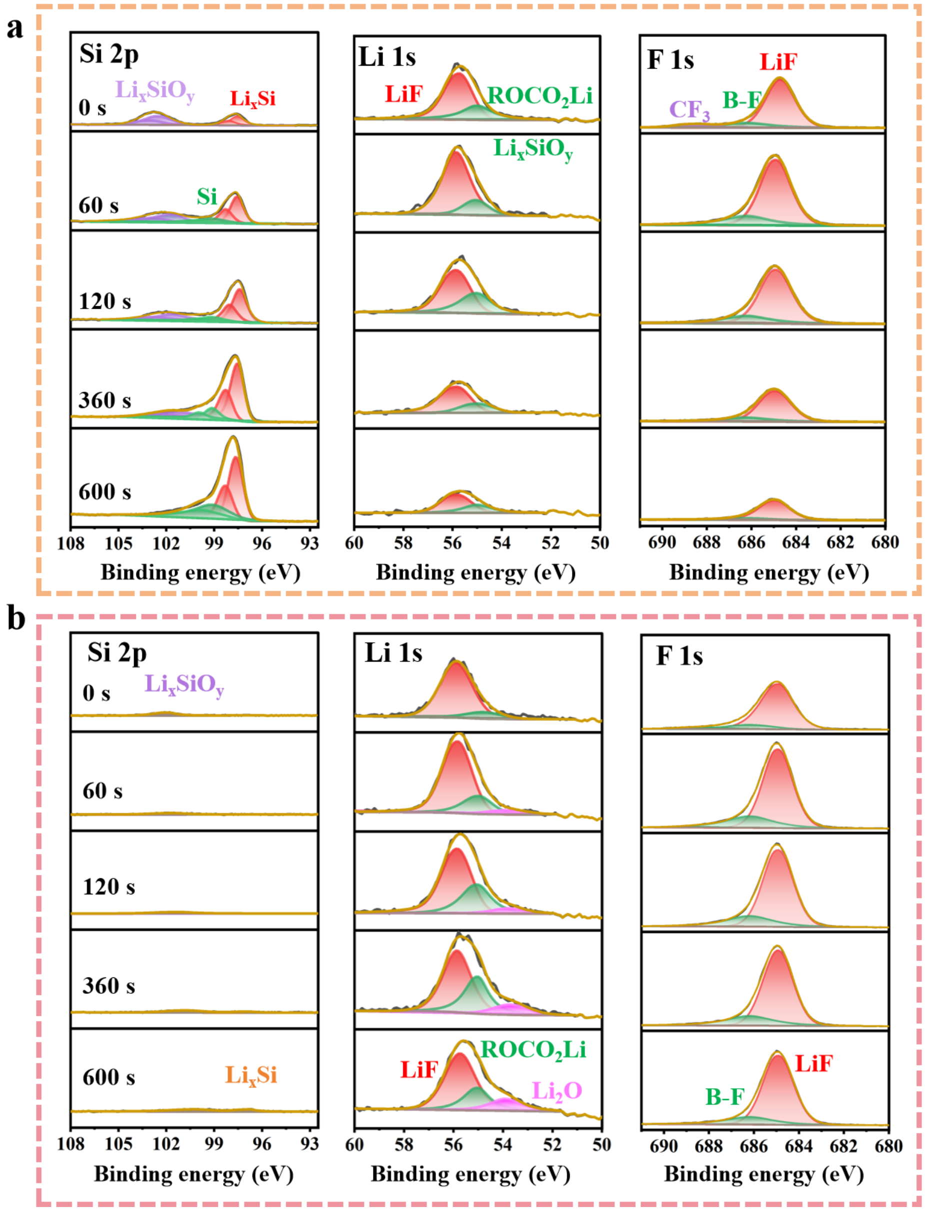

Figure S24. Si 2p, Li 1s and F 1s XPS patterns with Ar^+ sputtering of col-Si (**a**) and PL150-Si anode (**b**) after 3 cycles in coin-type full-cells.

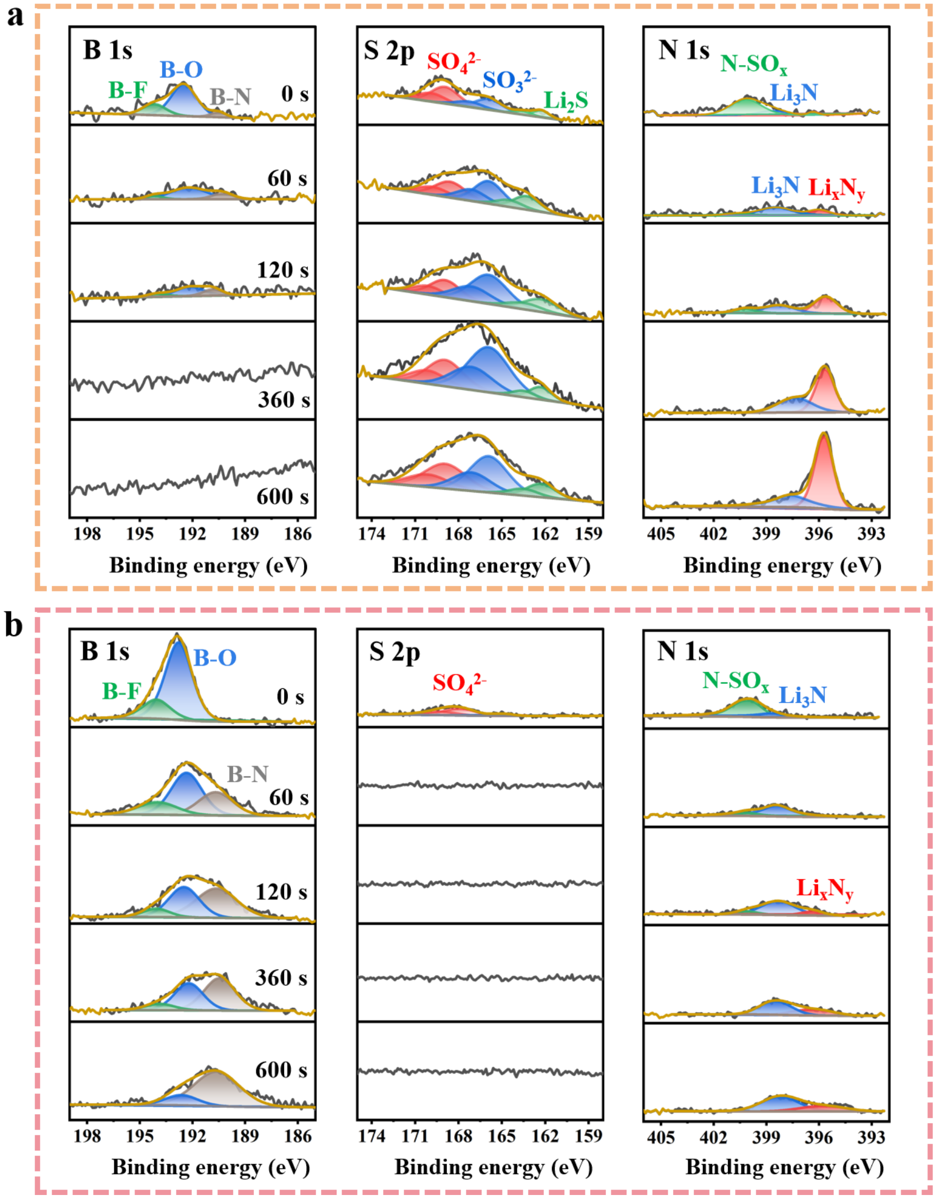

Figure S25. B 1s, S 2p and N 1s XPS patterns with Ar^+ sputtering of col-Si (**a**) and PL150-Si anode (**b**) after 3 cycles in coin-type full-cells.

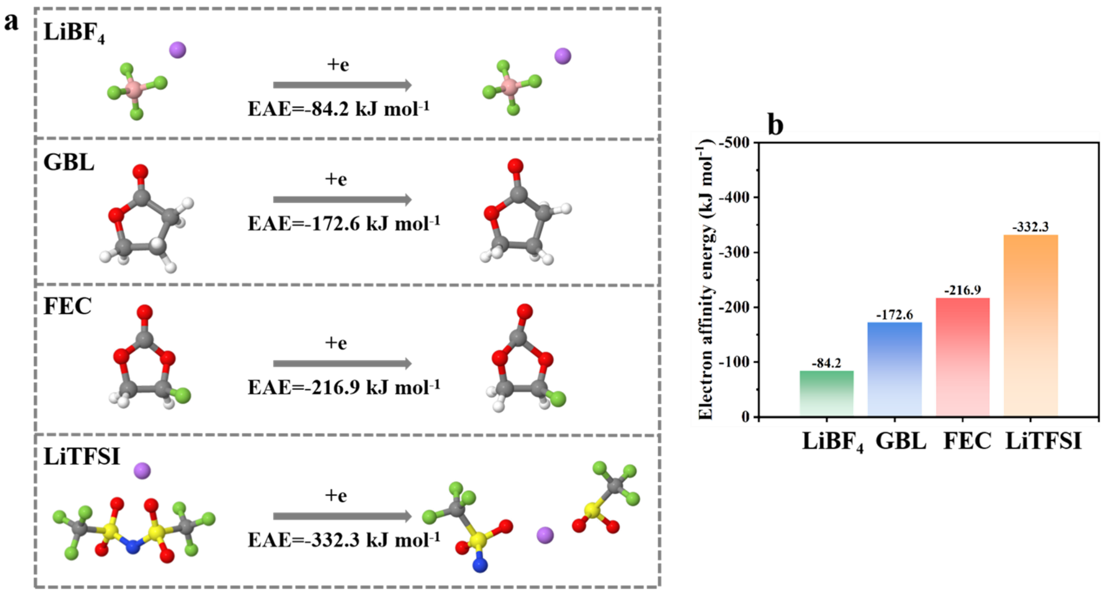

Figure S26. Optimized structures of four molecules in liquid electrolytes before and after single-electron reduction (**a**) and comparison of electron affinity energy (EAE) of these molecules (**b**).

Table S1. Literature summary of current methods for prelithiation of silicon-based anode and their impact on ICE and cycling performance in full-cell fabrication [6-26].

Anode materials	Prelithiation method	full-cell type, cathode	ICE before, and after prelithiation	Capacity retention, cycle number, current rate (after prelithiation)	Ref.
Si/C	direct contact with lithium metal	coin, $LiNi_{0.8}Co_{0.1}Mn_{0.1}O_2$	83.0%, 89.2%	95.1%, 98, 0.1C	[6]
Si (SiOx)	direct contact with lithium metal	coin, $LiFePO_4$	79%, 94% (44%, 94%)	44%, 200, C/3 (77%, 200, C/3)	[7]
Si/graphite	direct contact with lithium metal deposited on the Si-graphite electrode through thermal evaporation	coin, $LiCoO_2$	76.4%, 92.5%	80%, 366, 0.3C	[8]
Si	direct contact with lithium metal deposited on the Si electrode through thermal evaporation	coin, $LiNi_{0.8}Co_{0.1}Mn_{0.1}O_2$	72%, 85%	77%, 150, 0.33C	[9]
Si	direct contact with SLMP	coin, $LiNi_{0.8}Co_{0.1}Mn_{0.1}O_2$	—	87%, 80, 0.1C	[10]
SiO	direct contact with SLMP	coin, $LiFePO_4$	—	<50%, 100, 0.2C	[11]
SiOx/C	direct contact with SLMP	coin, $LiFePO_4$	98% (after)	78%, 100, 0.5C	[12]
Si	direct contact with Li-CNT film	coin, $LiNi_{0.8}Co_{0.1}Mn_{0.1}O_2$	78%, 88%	97.3%, 53, 0.5C	[13]
Si/graphite	Indirect contact with lithium metal through a intermediate buffer layer	pouch, $LiNi_{0.8}Co_{0.1}Mn_{0.1}O_2$	71%, 88.6%	86.3%, 1000, 0.5C	[14]
SiOx	Indirect contact with lithium metal through a intermediate buffer layer	coin, $LiNi_{0.6}Co_{0.2}Mn_{0.2}O_2$	68%, 87%	77%, 200, 0.5C	[15]
SiOx/graphite	active material (LiH)	pouch, $LiNi_{0.6}Co_{0.2}Mn_{0.2}O_2$	59.0%, 88.1%	74%, 800, 1C	[16]
Si/SiOx	active material ($LiBH_4$)	coin, $LiFePO_4$	91.1% (after)	83.6%, 100, 1C	[17]
SiOx	electrochemical prelithiation	coin, $LiNi_{0.8}Co_{0.15}Al_{0.05}O_2$	58.9%, 85.3%	61%, 100, 1C	[18]
Si/C	chemical prelithiation (1 M Li-biphenyl-DME)	coin, $LiNi_{0.8}Co_{0.15}Al_{0.05}O_2$	80.5%, 86.8%	76.5%, 200, 0.5C	[19]
SiO/graphene	chemical prelithiation (1 M Li−9,9-dimethyl-9H-fluorene−THF)	coin, $LiNi_{0.6}Co_{0.2}Mn_{0.2}O_2$	61.1%, 87.1%	80%, 100, 0.5C	[20]
SiOx/graphite	chemical prelithiation (0.2 M Li-biphenyl-2-methyl-trahydrofuran)	coin, $LiNi_{0.6}Co_{0.2}Mn_{0.2}O_2$	65.1%, 88.3%	87.3%, 250, 0.25C	[21]
SiO/C	chemical prelithiation (1 M 4-methylbiphenyl-2-methyl-trahydrofuran)	coin, $LiCoO_2$	40.9%, 83.7%	83.4%, 35, <1C	[22]
SiOx	chemical prelithiation (1 M naphthalene-2-methyl-tetrahydrofuran)	coin, $Li_{1.14}Ni_{0.13}Mn_{0.13}Co_{0.54}O_2$	64.6%, 84.4%	99.3%, 100, 1C	[23]
SiOx/C	chemical prelithiation (0.5 M Li-biphenyl-2-methyl-trahydrofuran)	coin, $LiNi_{0.6}Co_{0.2}Mn_{0.2}O_2$	47.8%, 85.7%	46%, 100, 0.2C	[24]
Siloxene	chemical prelithiation (0.5 M Li-4,4′- dimethylbiphenyl-DME)	coin, $LiNi_{0.5}Mn_{1.5}O_2$	—	94.3%, 200, <1C	[25]
SiOx/C@graphite	chemical prelithiation (1 M Li-biphenyl-THF)	pouch, $LiNi_{0.8}Co_{0.1}Mn_{0.1}O_2$	86%(after)	93.3%, 100, 0.2C	[26]
Si	chemical prelithiation (0.5 M Li-naphthalene-DME)	pouch, LMFP&LMO	74%, 96.6%	90.1%, 800, 1C	this work

Table S2. Comparison of electrical resistivity and electrical conductivity of n-Si, m-Si and col-Si.

Anode	Thickness of the Si layer	Test point	Electrical resistivity (Ω cm)	Average electrical resistivity (Ω cm)	Electrical conductivity (S cm^{-1})	Average electrical conductivity (S cm^{-1})
Nano-sized Si (n-Si)	17 μm	1	6.27E-06	6.27E-06	1.59E+05	1.60E+05
		2	6.6E-06		1.52E+05	
		3	5.94E-06		1.68E+05	
Micron-sized Si (m-Si)	10 μm	1	4.68E-06	4.94E-06	2.14E+05	2.03E+05
		2	5.2E-06		1.92E+05	
		3	4.94E-06		2.02E+05	
Col-Si (PVD)	5 μm	1	0.32	0.46	3.125	2.364
		2	0.64		1.563	
		3	0.416		2.404	

References:

1. Li Y, Cao Z, Wang Y *et al.* New insight into the role of fluoro-ethylene carbonate in suppressing Li-trapping for Si anodes in lithium-ion batteries. *ACS Energy Lett* 2023; **8**: 4193-203.

2. Quan L, Su Q, Wu H *et al.* All-climate outstanding-performances lithium-ion batteries enabled by in-situ constructed gel polymer electrolytes. *Chem Eng J* 2023; **454**: 140086.

3. Li Z, Lu Y, Su Q *et al.* High-power bipolar solid-state batteries enabled by in-situ-formed ionogels for vehicle applications. *ACS Appl Mater Interfaces* 2022; **14**: 5402-13.

4. Liu M, Vatamanu J, Chen X *et al.* Hydrolysis of $LiPF_6$-containing electrolyte at high voltage. *ACS Energy Lett* 2021; **6**: 2096-102.

5. Wang Y, Xing L, Li W *et al.* Why do sulfone-based electrolytes show stability at high voltages? Insight from density functional theory. *J Phys Chem Lett* 2013; **4**: 3992-9.

6. Yang C, Ma H, Yuan R *et al.* Roll-to-roll prelithiation of lithium-ion battery anodes by transfer printing. *Nat Energy* 2023; **8**: 703-13.

7. Bai S, Bao W, Qian K *et al.* Elucidating the role of prelithiation in Si‐based anodes for interface stabilization. *Adv Energy Mater* 2023; **13**: 2301041.

8. Kim K H, Shon J, Jeong H *et al.* Improving the cyclability of silicon anodes for lithium-ion batteries using a simple pre-lithiation method. *J Power Sources* 2020; **459**: 228066.

9. Adhitama E, Bela M M, Demelash F *et al.* On the practical applicability of the Li metal‐based thermal evaporation prelithiation technique on Si anodes for lithium ion batteries. *Adv Energy Mater* 2022; **13**: 2203256.

10. Jang E, Ryu S, Kim M *et al.* Silicon-stabilized lithium metal powder (SLMP) composite anodes for fast charging by in-situ prelithiation. *J Power Sources* 2023; **580**: 233326.

11. Huang B, Huang T, Wan L *et al.* Pre-lithiating SiO anodes for lithium-ion batteries by a simple, effective, and controllable strategy using stabilized lithium metal powder. *ACS Sustainable Chem Eng* 2021; **9**: 648-57.

12. Sun Q, Li J, Hao C *et al.* Focusing on the subsequent coulombic efficiencies of SiO_x: initial high-temperature charge after over-capacity prelithiation for high-efficiency SiO_x-based full-cell battery. *ACS Appl Mater Interfaces* 2022; **14**: 14284-92.

13. Wang C, Yang F, Wan W *et al.* A large-area lithium metal–carbon nanotube film for precise contact prelithiation in lithium-ion batteries. *Energy Environ Sci* 2023; **16**: 4660-9.

14. Wang H, Zhang M, Jia Q *et al.* Exploiting the capacity merits of Si anodes in the energy-dense prototypes via a homogeneous prelithiation therapy. *Nano Energy* 2022; **95**: 107026.

15. Meng Q, Li G, Yue J *et al.* High-Performance Lithiated SiOx Anode Obtained by a Controllable and Efficient Prelithiation Strategy. *ACS Appl Mater Interfaces* 2019; **11**: 32062-8.

16. Chung D J, Youn D, Kim S *et al.* Dehydrogenation-driven Li metal-free prelithiation for high initial efficiency SiO-based lithium storage materials. *Nano Energy* 2021; **89**: 106378.

17. Zhu Y, Hu W, Zhou J *et al.* Prelithiated surface oxide layer enabled high-performance Si anode for lithium storage. *ACS Appl Mater Interfaces* 2019; **11**: 18305-12.

18. Kim H J, Choi S, Lee S J *et al.* Controlled prelithiation of silicon monoxide for high performance lithium-ion rechargeable full cells. *Nano Lett* 2015; **16**: 282-8.

19. Kim K H, Nam M G, Kim M J *et al.* Selective incorporation of inorganic solid-electrolyte interphase into carbon-coated silicon nanoparticle anode for lithium-ion batteries with improved cyclic stability. *Carbon* 2023; **210**: 118056.

20. Zhang X, Qu H, Ji W *et al.* An electrode-level prelithiation of SiO anodes with organolithium compounds for lithium-ion batteries. *J Power Sources* 2020; **478**: 229067.

21. Choi J, Jeong H, Jang J *et al.* Weakly solvating solution enables chemical prelithiation of graphite–SiO_x anodes for high-energy Li-ion batteries. *J Am Chem Soc* 2021; **143**: 9169-76.

22. He W, Xu H, Chen Z *et al.* Regulating the solvation structure of Li^+ enables chemical prelithiation of silicon-based anodes toward high-energy lithium-ion batteries. *Nano-Micro Lett* 2023; **15**: 107.

23. Li X, Bian C, Zhang J *et al.* Chemical pre-lithiation of SiO_x anodes with a weakly solvating solution of polycyclic aromatic hydrocarbons for lithium-ion batteries. *ACS Appl Energy Mater* 2023; **6**: 8919-28.

24. Jiang F, Sun Y, Zhang K *et al.* SiO_x/C anodes with high initial coulombic efficiency through the synergy effect of pre-lithiation and fluoroethylene carbonate for lithium-ion batteries. *Electrochim Acta* 2021; **398**: 139315.

25. Shen H, An Y, Man Q *et al.* Controlled prelithiation of siloxene nanosheet anodes enables high performance 5 V-class lithium-ion batteries. *Chem Eng J* 2023; **454**: 140136.

26. Yan M, Li G, Zhang J *et al.* Enabling SiO_x/C anode with high initial coulombic efficiency through a chemical pre-lithiation strategy for high-energy-density lithium-ion batteries. *ACS Appl Mater Interfaces* 2020; **12**: 27202-9.
